# Conflict over condition-dependent sex allocation can lead to mixed sex-determination systems

**DOI:** 10.1111/evo.12513

**Published:** 2014-10-20

**Authors:** Bram Kuijper, Ido Pen

**Affiliations:** 1 Theoretical Biology Group, Centre for Evolutionary and Ecological Studies, University of Groningenthe Netherlands; 2 ComPLEX, Centre for Mathematics and Physics in the Life Sciences and Experimental Biology, University College LondonLondon, United Kingdom; 3 Department of Genetics, Evolution and Environment, University College LondonLondon, United Kingdom

**Keywords:** Genetic conflict, heterogamety, parent–offspring conflict, sex chromosomes, sex determination, sex ratios, temperature

## Abstract

Theory suggests that genetic conflicts drive turnovers between sex-determining mechanisms, yet these studies only apply to cases where sex allocation is independent of environment or condition. Here, we model parent–offspring conflict in the presence of condition-dependent sex allocation, where the environment has sex-specific fitness consequences. Additionally, one sex is assumed to be more costly to produce than the other, which leads offspring to favor a sex ratio less biased toward the cheaper sex in comparison to the sex ratio favored by mothers. The scope for parent–offspring conflict depends on the relative frequency of both environments: when one environment is less common than the other, parent–offspring conflict can be reduced or even entirely absent, despite a biased population sex ratio. The model shows that conflict-driven invasions of condition-independent sex factors (e.g., sex chromosomes) result either in the loss of condition-dependent sex allocation, or, interestingly, lead to stable mixtures of condition-dependent and condition-independent sex factors. The latter outcome corresponds to empirical observations in which sex chromosomes are present in organisms with environment-dependent sex determination. Finally, conflict can also favor errors in environmental perception, potentially resulting in the loss of condition-dependent sex allocation without genetic changes to sex-determining loci.

Condition-dependent sex allocation—where investment in one sex versus the other is dependent on the environment or an individual's condition—provides an adaptation to environments that have different fitness consequences for males and females (Trivers and Willard 1973; Charnov and Bull 1977). Studies on a number of taxa have shown that environments that are more beneficial to males than females lead to the overproduction of sons, whereas the reverse condition leads to the overproduction of daughters (see West 2009, and references therein). Prominent examples are the sensitivity of sex-specific fitness to developmental temperature in lizards, associated with environment-dependent sex determination (ESD) based on temperature (Warner and Shine 2008; Pen et al. 2010) or facultative sex ratios based on host size in parasitoid wasps (Charnov et al. 1981). However, results are not always that straightforward, with facultative sex ratios being strikingly absent in other taxa, despite clear indications that male and female fitness differentially depends on the environment (e.g., Hewison and Gaillard 1999; Rutstein et al. 2005; Uller and Olsson 2006). Hence, the factors that underlie the evolutionary maintenance of condition-dependent sex allocation are still poorly understood (West 2009).

An interesting observation arising from phylogenetic studies is that transitions between ESD and genetic sex determining systems (GSD) are relatively rapid, in which closely related species (Janzen and Phillips 2006; Mank et al. 2006; Quinn et al. 2011; Sarre et al. 2011) and sometimes even different local populations of the same species (Pen et al. 2010) have diverged in their mode of sex determination. Such evolutionary transitions between ESD and GSD are currently exclusively ascribed to environmental change, such as changes in climate (e.g., Pen et al. 2010; Grossen et al. 2011). This focus on the role of the environment overlooks, however, important insights from studies on genetic sex determination, which highlight that genetic conflicts over sex allocation drive transitions between sex-determining mechanisms (e.g., Rigaud and Juchault 1993; Werren and Beukeboom 1998; Werren et al. 2002; Uller et al. 2007; Van Doorn and Kirkpatrick 2007; Cordaux et al. 2011). To our knowledge, however, no work has been done on the role of genetic conflicts in the context of condition-dependent sex allocation.

To address this gap, we focus on a model of parent–offspring conflict (or more specifically, mother–offspring conflict) over condition-dependent sex allocation. Evolutionary interests between mothers and offspring over sex allocation can diverge, because mothers are typically equally related to all their offspring and therefore maximize their fitness by producing a sex ratio that maximizes the total reproductive value of her current and future broods. In contrast, individual offspring are more closely related to themselves than to their siblings, and may therefore prefer to develop as the rarer sex with a higher reproductive value, at the expense of their siblings (Trivers 1974; Eshel and Sansone 1991). Such conflicts between parents and offspring over sex allocation have already been associated with transitions in female and male heterogamety in the context of GSD (Werren et al. 2002; Pen 2006; Kozielska 2008), but whether parent–offspring conflict can also play a role in transitions between condition-dependent and condition-independent sex-determining mechanisms is currently unclear.

To model the interaction between parent–offspring conflict and condition-dependent sex allocation, we focus on the seminal Charnov–Bull model (e.g., Charnov and Bull 1977; Schwanz et al. 2006), which assumes that mothers encounter one of two environmental conditions, one of which reduces the fitness (here juvenile survival) of one sex. For example, mothers could breed in different environments, one of which benefits male development more than female development. In case of condition-independent sex determination, individuals would then often develop as the “wrong sex,” whereas condition-dependent sex determination limits the production of that sex in the environment to which it is maladapted (e.g., Trivers and Willard 1973; Charnov and Bull 1977; Bull 1981; Bull and Bulmer 1989). Here, we analyze equilibrium sex-allocation strategies when condition-dependent sex allocation is either controlled by the mother or by the offspring. Using an analytical reproductive value approach (Leimar 1996; Taylor 1996) in combination with individual-based simulations, we then assess the conditions in which maternal and offspring sex-allocation strategies diverge (i.e., the conflict “battleground,” Godfray 1995).

Subsequently, we study a number of evolutionary resolutions to the conflict. Models in which sex determination is independent of condition have shown that parent–offspring conflict can pave the way for the invasion by genetic sex modifiers, which allows parents to achieve brood sex ratios closer to their optimal sex allocation, or allows offspring to develop more often as the sex with the higher reproductive value (Werren et al. 2002; Pen 2006; Kozielska 2008). Here, we investigate whether condition-independent genetic masculinizers or feminizers are able to invade in populations with condition-dependent sex allocation. In addition, we assess whether conflict over sex allocation may also favor the invasion by modifiers that change the sensitivity to environmental cues—on the basis of which sex is determined—to bring the sex-allocation optimum closer to either the offspring's or parental optimum.

## The Model

We consider a panmictic, monogamous sexual population with discrete generations. Similar to the seminal Charnov–Bull model (e.g., Charnov and Bull 1977; Schwanz et al. 2006), we assume that environmental variation has different fitness consequences to each sex. Specifically, with probability *p*, the mother breeds in the so-called poor environment (environment 1), which is detrimental to newborn daughters (which have survival *v*) relative to sons. With probability 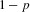, the mother breeds in a good environment (environment 2), in which juvenile survival is not sex-specific. For the sake of simplicity and in accordance to previous models (e.g., Charnov and Bull 1977; Wild and West 2007), we assume that only the environment in which a mother breeds affects her sex-allocation decision.

Additionally, we assume that the production of a son requires *c* units of maternal resources relative to each unit invested in daughters, where all mothers have accumulated an identical amount of resources. Sex ratios in the poor and good environments, respectively, are given by the strategy 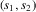. These sex ratios 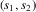 can either be expressed in the mother (subscript “m”) or expressed in the offspring/zygote (subscript “o”). For the sake of simplicity, we assume that the gene locus coding for 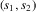 is haploid.

Overall, the life cycle is as follows: (1) birth and sex determination of offspring in the natal environment 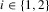, (2) environment and sex-specific juvenile survival to adulthood, (3) random settlement of mothers in one of two environments, (4) random mating with those males who dispersed to the maternal breeding environment, and (5) reproduction, after which all adults die. Subsequently, the cycle repeats again with the birth of offspring. Note that the timing of male dispersal may affect the evolution of condition-dependent sex allocation: in case male dispersal occurs after mating, males achieve all their reproductive success in their natal environment, after which they disperse but have no further reproductive opportunities. In this case, condition-dependent sex allocation will not evolve (see Section S1 of the Supporting Information). This is because an individual male's reproductive success will always be affected by a single environment (the natal environment), which eliminates one of the basic assumptions of the Charnov–Bull model, namely that any individual is likely to experience a certain variation in environmental conditions (Charnov and Bull 1977) (see also Fig. 3 in Wild and West 2007 for similar results). By contrast, when dispersal occurs before mating, any individual male encounters one or the other environment with a certain probability, say *d*_1_ (see Section S1 of the Supporting Information). When this probability is 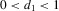, condition-dependent sex allocation evolves (it can be shown that sex-allocation optima are, in fact, independent of the magnitude of *d*_1_). We therefore focus on the case of dispersal before mating in the current study, as this is favorable to the evolution of condition-dependent sex allocation.

We implement a model for the evolution of condition-dependent sex determination using a reproductive value approach (Taylor 1996; Pen and Weissing 2000; Fawcett et al. 2011). The population consists of three classes of individuals: (1) females living in environment 1, (2) females living in environment 2, and (3) males living in both environments. Let 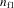 then describe the number of copies of the allele coding for sex-allocation strategy 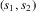 that are present in females who breed in environment 1. Similarly, 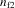 describes the number of copies of the 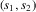 allele present in females who breed in environment 2, and lastly, *n*_m_ describes the number of 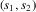 alleles present in males. We then consider a population that is monomorphic for the 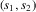 allele, so that the dynamic 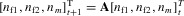 (T denoting transposition) tracks the number of gene copies passed on to the next generation. **A** is a matrix that governs transitions between the three different classes:1
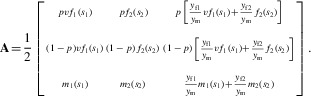
Note that the transition matrix **A** is multiplied by 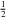, reflecting the genetic share of each parent in its offspring. The functions 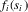 and 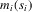 represent the number of females and males produced by a mother that breeds in environment *i*, using resident strategy 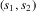. For the moment, we assume that mothers and offspring always correctly perceive the maternal breeding environment, but we relax this assumption later (see Section S7 in the Supporting Information). Because a son is *c* times more costly to produce than a daughter, the average amount of resources *K* invested per offspring in environment *i* is proportional to 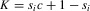, where *c* reflects, for example, the amount of calories invested in a son relative to the amount invested in a daughter. Similar to classical life-history models (Smith and Fretwell 1974), we assume that the total number of offspring is inversely proportional to the investment in each offspring, leading to the following expressions for the number of 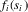 daughters and 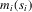 sons who are produced in environment *i*
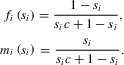
We explain some of the entries of the transition matrix **A** to clarify the setup of our model. The top-left entry *a*_11_ describes the contribution of females who breed in environment 1 at time *t* to females breeding in environment 1 at time 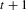. Adult females in environment 1 produce 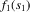 daughters. Because these daughters are born in environment 1, they have a reduced juvenile survival rate 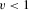, relative to sons born in the same environment and any offspring born in environment 2. A juvenile female subsequently has a probability *p* of breeding in environment 1 as an adult. The middle entry in the top row, *a*_12_, describes the contribution of females breeding in environment 2 at time *t* to females breeding in environment 1 at time 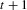. Because daughters grow up in environment 2, their survival probability is equal to 1, after which they settle with probability *p* in environment 1. The right entry in the top row, *a*_13_, describes the contribution of males at time *t* to females breeding in environment 1 at time 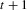. After birth, males are assumed to disperse to one of both breeding environments where they mate randomly with a female breeder. Consequently, the mating rate of a male with females breeding in environment 1 is given by the stable class frequency 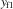 of females breeding in environment 1, divided by the stable class frequency of males 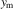. Subsequently, 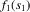 daughters are produced, who will survive in environment 1 with probability *v*. Alternatively, a male mates with a female breeding in environment 2 with rate 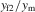, yielding 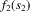 daughters who have a survival probability of 1. Subsequently, daughters sired by a male in environment 1 or 2 will breed in either environment with respective probabilities *p* and 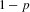. The entries in the other two rows can be derived in a similar fashion.

We are interested in the determining optimal sex-allocation strategy in each environment, 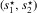. We do so by describing the number of gene copies passed on to the next generation by a rare mutant, with a slightly deviant sex-allocation strategy 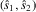, amidst a resident population that has sex-allocation strategy 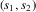. In the Appendix, we work out scenarios where either parents (section Maternal Control Over Sex Allocation) or offspring (section Offspring Control Over Sex Allocation) are in control over sex allocation. The class transitions of this rare mutant are given by the mutant transition matrix **B** (eqs. A1 and A10). Based on this mutant transition matrix, selection differentials acting on a particular trait 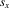 are obtained using a standard result (e.g., Taylor 1996)2
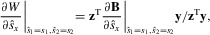
where **y** is a vector containing the stable class frequencies of the resident population (a dominant right eigenvector of matrix **B** evaluated at the resident behavior 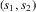), whereas **z** are the individual reproductive values (equal to a dominant left eigenvector of matrix **B** evaluated at the resident behavior 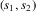). In case of maternal control over sex allocation, a mutant mother with sex-allocation strategy 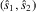 affects all members of the brood alike, so that the selection differential on each sex-allocation trait is given by3
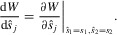
In case of offspring control over sex allocation, a mutant offspring's fitness is affected both by its own sex-allocation strategy 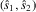 and the sex-allocation strategy 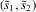 of its siblings (see Appendix). Using a direct fitness approach (Taylor and Frank 1996; Pen and Weissing 2002; Taylor et al. 2007), the selection differential on each sex-allocation trait is given by4
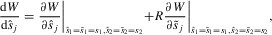
where *R* is the relatedness between a focal mutant offspring with a randomly chosen sibling, which is approximately 1/2 when broods are large and mothers mate only once. Explicit expressions for the selection differentials can be found in the Appendix.

We subsequently investigate whether there are equilibrium sex-allocation strategies by assessing when the selection differentials vanish. We find no equilibria where 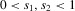. Therefore, the equilibria for one or both sex-allocation strategies 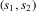 should reside at the boundaries 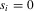 or 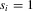, which is a well-known feature of the Charnov–Bull model (e.g., Schwanz et al. 2006; Wild and West 2007).

### INDIVIDUAL-BASED SIMULATIONS

In addition to the analytical model, we also ran stochastic individual-based simulations to corroborate analytical results. We modeled a population of 5000 individuals, each bearing two unlinked, diploid, autosomal genetic loci coding for *s*_1_ and *s*_2_, respectively. Mutation in the unlinked sex-allocation loci occurs following a continuum of alleles model, where each individual allele has a mutation rate 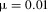. If an allele mutates, its value is incremented with a number drawn from a normal distribution with mean 0 and variance 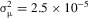. If the new allelic value lies outside the range (0, 1), it is set to its nearest value within that range (i.e., 0 or 1).

The life cycle mimics that of the analytical model: during each generation, females are randomly assigned to one of two environments with probability *p*. Subsequently, each female is assigned a mating partner that is randomly selected from the pool of males and offspring are produced. We assume that each female has a total amount of reproductive resources *r* that is equal to the cost of 50 sons. The sex of each offspring is determined randomly for each individual offspring, based on the sex-allocation locus that corresponds to the environment perceived by the individual controlling sex allocation (mother vs. offspring). With the production of each offspring, maternal resources are depleted by an amount 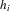 that is dependent on the sex of the offspring (son: 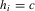, daughter: 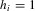) and offspring production is ceased when resources are equal to 0. In case the level of resources *r* is larger than zero, but less than the amount 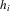 that is required for the production of the next offspring (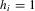 or 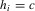), this offspring will only be produced if a random number drawn from a uniform distribution is smaller than 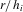. Subsequently, offspring survive dependent on their maternal environment and their sex. Adults that make up the next generation are randomly selected from the pool of surviving offspring. Simulations were coded in C and can be downloaded from the corresponding author's website.

### INVASION BY CONDITION-INDEPENDENT SEX FACTORS

We also assess whether parent–offspring conflict favors invasion by condition-independent sex factors (e.g., genetic sex determining loci), which could potentially lead to the replacement of condition-dependent sex allocation by sex-determining mechanisms that are independent of condition (such as genetic sex determination, GSD). We focus on four different scenarios of invasion: (1) invasion by a dominant masculinizer *Y* and (2) a feminizer *W* expressed by the offspring in populations where condition-dependent sex allocation is controlled by the mother, and the invasion by (3) brood masculinizers *M*_m_ and (4) feminizers *F*_m_ expressed by the mother in populations where condition-dependent sex allocation is controlled by offspring.

We assume that the sex ratio is the result of three unlinked loci *S*_1_, *S*_2_, and the genetic sex determining locus *G*, notation of which varies according to each of the four scenarios of invasion (see below). Loci *S*_1_ and *S*_2_ code for the sex-allocation reaction norm that gives rise to the condition-dependent sex-allocation strategy 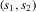 and are either expressed in the mother or offspring. Following the conventional adaptive dynamics approach, we assume that gene action at loci *S*_1_ and *S*_2_ is additive. Because a diploid locus with additive gene action is effectively functioning like a haploid locus, we assume for the sake of simplicity that *S*_1_ and *S*_2_ obey haploid inheritance (individual-based simulations assuming diploid loci reach similar results). Evolution at each locus 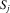 is then given by the dynamic in equation 3, assuming the successive invasion and substitution of condition-dependent sex-allocation mutants of small effect (Dieckmann and Law 1996; Geritz et al. 1998; Dercole and Rinaldi 2008).

In contrast to continuous evolution at loci *S*_1_ and *S*_2_, genetic variation at the diploid genetic sex determining locus *G* is discrete. Initially, only null alleles are present at *G*, which have no effect on sex allocation. We then consider the invasion by a dominant, condition-independent sex factor of large phenotypic effect that overrides *S*_1_ and *S*_2_. For each of the four scenarios of invasion, the invading sex factor is given by the following dominant alleles: (1) *Y*, whose presence in offspring always leads to male development, (2) *W*, whose presence in offspring always leads to female development, (3) *M*_m_, whose presence in mothers leads them to produce all-male broods, (4) *F*_m_, whose presence in mothers leads them to produce all-female broods.

To track the changes in the frequency of the alleles present at locus *G*, we change the transition matrix **B** in equation 1, so that the different classes of individuals now reflect the male or female bearers of the different genotypes (transition matrices for each of the four invasion scenarios are presented in the Supporting Information). Here, we discuss the example in which a novel *Y* chromosome whose presence in offspring always leads to male development. *Y* invades in a population in which all individuals bear the null-allele *y*, and which therefore exhibit maternally controlled condition-dependent sex allocation given by sex-allocation loci *s*_1_ and *s*_2_. We have four phenotypic classes: 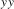 females living in environment 1 that are homozygous for the null allele (frequency *x*_1_), *yy* females living in environment 2 that are homozygous for the null allele (frequency *x*_2_), *yy* males living in either environment that are homozygous for the null allele (frequency *x*_3_), and *Yy* males (with frequency *x*_4_). Note that 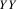 males do not exist, as *Yy* males necessarily mate with *yy* females. Consequently, evolutionary change in the frequency of the *Yy* genotype is given by the population genetics recursion5
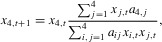
where 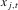 reflects the frequency of each of the different phenotypic classes and 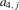 reflects the number of 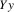 individuals produced by individuals of phenotypic class *j*, which are the corresponding entries in the resident transition matrix 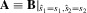. Similar recursions are obtained for all genotypes in the scenarios, involving the invasion by *W*, *F*_m_, or *M*_m_ in the Supporting Information.

Initial invasion by the 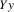 haplotype takes place in a population that is fixed for 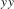 and that therefore exhibits a resident maternal sex allocation strategy 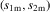 in Table 1. For the sake of tractability, we assume a separation of timescales, where upon successful invasion, the condition-independent genotype 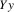 reaches its equilibrium frequency. Subsequently, we update the reproductive values and allow a condition-dependent sex-allocation mutant 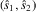 of small effect at either the *S*_1_ or *S*_2_ locus to invade and become the new resident maternal sex allocation phenotype. After that, we again update the reproductive values and then allow the condition-independent genotype 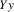 to achieve a new equilibrium frequency. We repeat these steps until both the frequency of the condition-independent genotype and the values of condition-dependent sex-allocation strategies remain unchanged. In addition, we ran individual-based simulations, in which no such separation of timescales was assumed, and which reach very similar outcomes. In the results, we therefore only present the individual-based simulations. The numerical iterations for all four scenarios can be downloaded from the corresponding author's website.

**Table 1:** Equilibrium condition–dependent sex ratios in case sex-allocation strategies are expressed by mother versus offspring

Region	I	II	III
**Expressed in mother**			
Boundaries	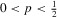	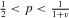	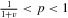
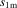	1	1	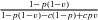
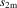	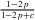	0	0
			
Population sex ratio (% males)	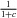	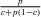	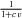
**Expressed in offspring**			
Boundaries	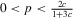	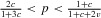	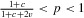
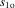	1	1	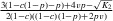
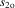	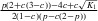	0	0
Population sex ratio (% males)	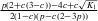	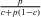	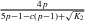
Derivatives	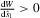 , 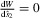	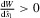 , 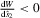	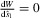 , 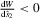

1 Regions I, II and III correspond to different combinations of pure and mixed sex strategies of the loci 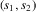, depicted in Figure 1. For sake of brevity, the parameters *K*_1_ and *K*_2_ reflect the contents of the square roots of the sex-allocation strategies under offspring control: 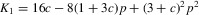, 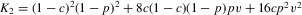.

## Results

To fix ideas, we first assess the extent of parent–offspring conflict over the sex ratio when sex allocation is independent of the environment or condition. To do so, we substitute for 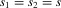 in equation 1 and derive the corresponding selection differential 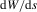 according to the Appendix. Solving for 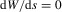, we obtain the classical sex-allocation equilibria for maternal control *s*_m_ (Fisher 1930) versus offspring control *s*_o_ (Trivers 1974)6
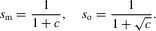
In other words, as soon as one sex is more costly to produce than the other (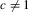), parents and offspring sex ratio optima diverge, as offspring favor a sex ratio that is less biased toward the cheaper sex than the sex ratio favored by parents (see also Fig. S1 for a graphical depiction of parental and offspring sex ratio equilibria for different values of *c*).

### DIVERGENCE OF MATERNAL AND OFFSPRING CONDITION-DEPENDENT SEX ALLOCATION

For a scenario in which sex allocation is dependent on condition, Table 1 compares sex-allocation equilibria for maternal versus offspring control over sex allocation. To facilitate interpretation, a graphical example of maternal and offspring sex allocation equilibria is given in Figure 1. Qualitative outcomes of our model are similar to previous analyses of the classical Charnov–Bull model (e.g., Schwanz et al. 2006; Wild and West 2007). For both parents and offspring, one or both sex ratios 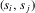 must always be at a boundary (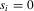 and/or 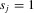, see Appendix), leading to three qualitatively different regions (denoted by I, II, and III in Fig. 1 and Table 1).

**Figure 1:**
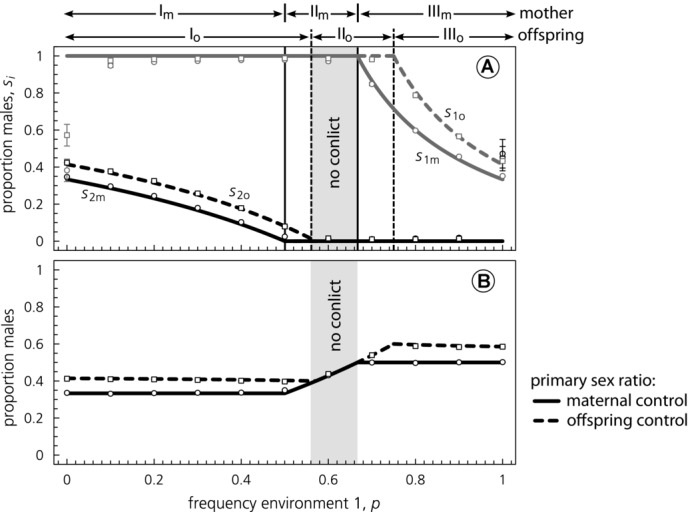
A graphical depiction of the analytically obtained maternal (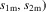 (solid lines) and offspring (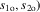 (dotted lines) sex-allocation optima from Table 1 when sons are twice as costly to produce than daughters (panel A) and the resulting population-wide primary sex ratios under maternal and offspring control (panel B). The different regions I, II, III from Table 1 for maternal and offspring control are depicted by the arrows on top of panel A. Parent–offspring conflict exists in the white regions, where offspring sex allocation is less biased toward the cheaper and hence rarer sex. Interests of parents and offspring converge in the middle gray region. The analytical results are confirmed by individual-based simulations, of which the mean sex-allocation strategies 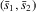 averaged over 10 replicate individual-based simulations after 70, 000 generations are indicated by circles (maternal control) and squares (offspring control), with bars indicating standard errors (which are generally small). Parameters: 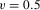.

Although sex differences in survival *v* affect the extent of conflict over sex allocation between parents and offspring (e.g., see Fig. S1), *v* by itself does not give rise to conflict. We find that also in the condition-dependent case, differences between the sexes in their production costs 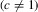 are essential for parental 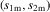 and offspring sex allocation equilibria to diverge 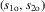. As the current model considers a well-mixed population, it is unsurprising that a sex difference in survival *v* has no effect on conflict when 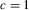, as in this case any deviant sex allocation by a focal offspring does not affect the redistribution of resources among the brood. By contrast, whenever 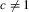, a single offspring's sex allocation decision immediately affects the total number of siblings produced in the remainder of the brood, thus giving rise to parent–offspring conflict (Trivers 1974; Werren et al. 2002). In the following, we therefore discuss parent–offspring conflict for an example case in which sons are twice as costly as daughters (i.e., 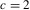) and where female juveniles born in environment 1 have a lowered survival 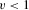 (see Fig. 1):

1. In region I, the poor environment 1 is relatively rare. Consequently, individuals are selected to avoid producing any females in the poor environment 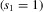, while overproducing females in the good environment 2. Compared to their mothers, offspring always favor a more male-biased sex ratio in the good environment, because sons are the more costly (and hence rarer) sex (see Fig. 1 B), therefore having a higher reproductive value (Trivers 1974). Hence, 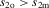.
2. Region II: when the poor environment 1 is more prevalent, individuals in that environment still exclusively produce males, but individuals in the good environment 2 now exclusively produce females (a “bang-bang” sex-allocation strategy, 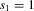, 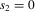). Parents and offspring have, however, a different range of frequencies *p* in which they selectively favor a bang-bang sex-allocation strategy. This is because
  Female-biased population sex ratios select for some offspring to develop themselves as males in environment 2, whereas their mothers favor the exclusive production of daughters in the latter environment. Nonetheless, for a range of frequencies, both parents and offspring are in agreement by favoring a bang-bang sex-allocation strategy (gray areas in Fig. 1), so that conflict is absent despite the presence of sex-specific production costs.
3. Region III: when the poor environment 1 is very common, the bang-bang sex-allocation strategy is replaced by a mixed sex ratio in environment 1 (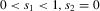). Because males are, however, still overproduced in environment 1, the overall population sex ratio becomes more male biased in comparison to population sex ratios in regions I and II (Fig. 1 B), which is in line with classical predictions (Bull and Charnov 1988; Frank and Swingland 1988) that sex ratios should be biased toward the sex overproduced in the poor environment (males). Although such male-biased population sex ratios potentially reduce the reproductive value of males, we find that sex differences in production costs *c* are still sufficient to have offspring prefer an even more male-biased sex ratio than their parents (see Fig. 1 B).

To summarize, conflicts between parents and offspring over condition-dependent sex allocation are thus highly context-dependent, with divergent selective optima typically occurring in only one of both environments. Moreover, the extent of conflict is strongly dependent on the relative frequencies of both environments, where parent–offspring conflict is typically absent for an intermediate range of environmental frequencies. We now investigate whether these findings have ramifications for any evolutionary transitions between condition-dependent and condition-independent sex-determining mechanisms.

### CAN UNCONDITIONAL SEX DETERMINATION INVADE?

Following previous models, which showed that parent–offspring conflict can lead to the invasion and establishment of novel genetic sex factors (Werren et al. 2002; Kozielska 2008), we now analyze the invasion of populations with condition-dependent sex allocation by unconditional sex modifiers, such as sex chromosomes. As introduced in section “Invasion by Condition-Independent Sex Factors,” we focus on the invasion by four different condition-independent sex factors: (1) masculinizers expressed in offspring, (2) feminizers expressed in offspring, (3) brood feminizers expressed in the mothers, and (4) brood masculinizer expressed in mothers.

#### Invasion by unconditional sex factors expressed in the zygote

We focus here on the invasion by a masculinizing allele (hereafter: Y) expressed in the zygote, whereas corresponding results for the invasion by a feminizer *W* expressed in the zygote are given in Figure S4. Y invades in a population that is fixed for a null allele y, with the sex of yy individuals being determined according to maternal sex allocation loci 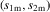, which have attained their condition-dependent sex-ratio equilibria as given in Table 1 and Figure 1. The Y allele is dominant, as heterozygous Yy individuals always develop as males. Similar to previous models (Werren et al. 2002; Pen 2006), the presence of Y also has an epistatic effect, as it overrides the maternally expressed sex-allocation loci 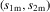. Note that YY individuals do not exist, as Yy males always mate with yy females.

**Figure 2:**
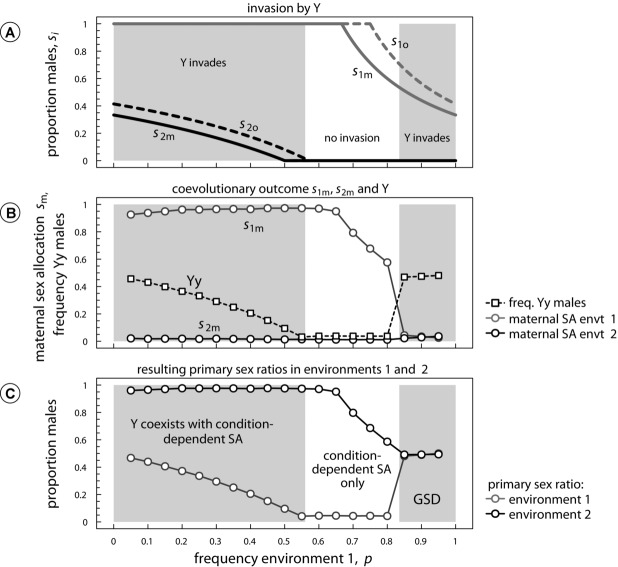
Invasion by a dominant masculinizer (Y) expressed in the zygote, when condition-dependent sex-allocation loci are expressed in the mother (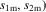 and when sons are twice as costly as daughters (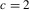). Panel A: parental and offspring sex allocation optima from Table 1, with gray areas depicting analytically obtained invasion conditions for Y (see also Fig. S2A). Panel B: the coevolutionary outcome between Y and maternal sex allocation strategies 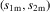 obtained from the individual-based simulations: as the frequency of males with genotype Yy increases, maternal sex allocation 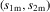 evolves to counter the overproduction of males, leading either to 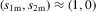 or 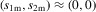. Panel C: primary sex ratios that result from the evolved frequency of Yy males and the values of *s*_1m_ and *s*_2m_ (obtained from the individual-based simulations), measured as the proportion of sons produced at birth in each environment. Despite the invasion by Y, the primary sex ratio still strongly depends on the maternal environment for a large range of environmental frequencies *p*, although resulting sex ratios are now closer to offspring than to parental optima. Only when the poor environment is highly prevalent (i.e., 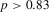), sex ratios are independent of the maternal environment, implying that the invasion by Y has led to a replacement of ESD by GSD. Parameters: 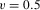. SA, sex allocation.

Unsurprisingly, invasion by Y is only possible when sons are more costly than daughters (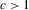, see also Fig. S2A), because this causes offspring to prefer a more male-biased sex ratio than their mothers. Additionally, the invasion by Y is dependent on the survival of females in the poor environment *v* and the frequency *p* of the poor environment. In particular, Figure 2 shows that for certain values of *p*, condition-dependent sex-allocation expressed by the mother is robust to the invasion by Y, despite offspring favoring a more male-biased sex ratio than their mothers in the poor environment (i.e., see white region in Fig. 2A where 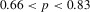). In this particular region, Y would benefit offspring in the poor environment by generating the desired more male-biased sex ratio. However, the presence of Y also results in the undesirable production of males in the good environment, where offspring favor to develop exclusively as females, 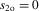. For those cases where 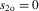, invasion by Y will therefore only ensue when environment 2 becomes sufficiently rare (Fig. 2A).

When Y is able to invade, coevolution between Y and maternal sex allocation loci 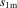 and 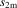 results in two qualitatively different outcomes: when the poor environment predominates (right side in Fig. 2), the invasion by Y is selectively favored by offspring in the poor environment. Mothers can only counter the male-biased sex ratios resulting from the invading Y by producing more daughters in the poor environment (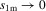), which in turn selects for ever higher frequencies of Y. Coevolution between Y and (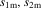) eventually leads to an equilibrium in which all individuals without a Y develop as females, as 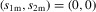. The frequency of Yy males is then equal to 0.5, as expected given the Mendelian necessity that half of all offspring inherit a Y chromosome when all males bear the Yy genotype. Consequently, conflict can lead to a replacement of condition-dependent sex allocation by GSD (i.e., male heterogamety), despite the fitness disadvantage caused by the production of daughters in the more common poor environment.

When the poor environment is less common (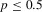), the invasion by Y is selectively favored by offspring in the good environment, whereas both parents and offspring in the poor environment 1 favor the exclusive production of sons (and hence are not affected by the invasion by Y). Subsequent to the invasion by Y, mothers in environment 2 are selected to produce ever more daughters from those offspring that did not receive Y (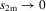) to counter the increased production of males in her brood. Figure 2B shows that coevolution between Y and 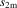 eventually halts when all offspring that do not carry a Y are maternally induced to become daughters 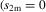), whereas Yy males reach frequencies close to the offspring sex allocation equilibrium 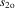 for environment 2 (in fact, sex ratios are marginally higher than 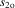 due to the highly discrete nature of Y, see Fig. S3A). As a result, the invasion by Y does not result in a transition from condition-dependent sex allocation expressed in the mother to condition-independent sex allocation. Rather, the presence of Y now facilitates offspring to achieve a pattern of sex allocation that is closer to the offspring optimum, so that condition-dependent sex allocation effectively shifts from maternal to offspring control (Fig. 2C). Interestingly, the offspring sex allocation phenotype is then the result of a “mixture” of sex-determining mechanisms, involving both genetic factors (Y) and condition-dependent sex-determining factors expressed in the mother.

#### Invasion by maternally expressed unconditional sex factors

We focus here on the invasion by a brood feminizer 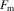 allele expressed in mothers (Werren et al. 2002), the presence of which leads to complete female development of a brood regardless of the environment. Maternal production of all-female broods irrespective of the environment has, for example, been observed in a number of arthropod taxa (e.g., White 1973; Ullerich 1984; Tabadkani et al. 2011). 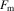 invades in a population that is otherwise fixed for a null allele f, where 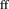 mothers defer control over sex allocation to offspring, who determine sex according to loci 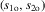. Before *F*_m_ invades, 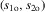 have attained their condition-dependent sex-allocation equilibria as given in Table 1 and Figure 1. The 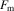 allele is dominant, as mothers with genotype 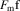 produce all-female broods. In addition, the presence of 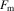 also has an epistatic effect, as it overrides the 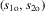 loci. Note that homozygous 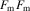 individuals do not exist, as 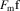 females always mate with 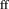 males. The model is presented in the Supporting Information, where we also derive an analogous case for maternal brood masculinizers 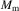 (e.g., see Fig. S5).

**Figure 3:**
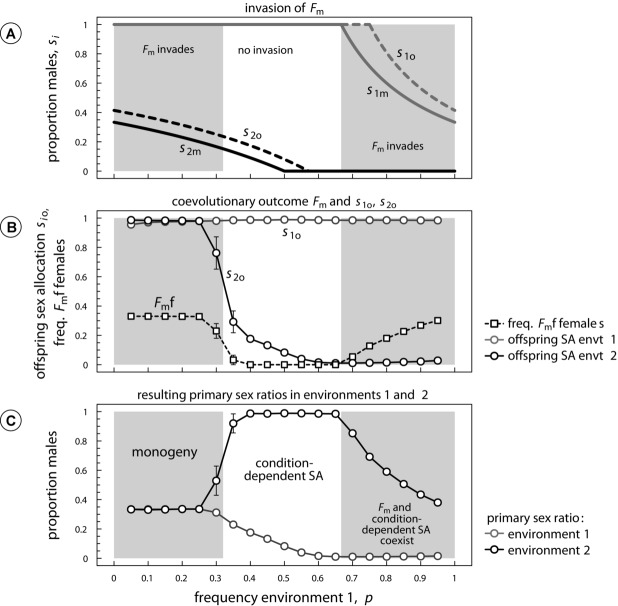
Invasion by a dominant feminizer (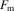) expressed by the mother, when condition-dependent sex-allocation loci are expressed by the zygote (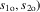 and when sons are twice as costly as daughters (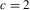). Panel A: parental and offspring sex allocation optima from Table 1, with gray areas depicting analytically obtained invasion conditions for *F*_m_. Panel B: the coevolutionary outcome between *F*_m_ and offspring sex-allocation strategies 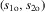 obtained from the individual-based simulations. As the frequency of mothers with genotype 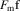 increases, offspring sex allocation becomes more male biased, leading either to 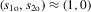 or 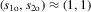. Note that due to the stochastic nature of the individual-based simulations, 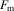 invades in a slightly wider range of conditions than predicted from the analytical invasion conditions. Panel C: primary sex ratios that result from the coevolved frequency of *F*_m_f females and values of offspring sex allocation 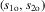 (obtained from the individual-based simulations). Primary sex ratios are measured as the proportion of sons produced at birth in each environment. Despite the invasion by *F*_m_, the primary sex ratio still strongly depends on the maternal environment for a large range of environmental frequencies *p*, although resulting sex ratios are now closer to maternal than offspring optima. Invasion by 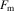 only leads to a replacement of condition-dependent sex allocation with monogeny when the poor environment is relatively rare (i.e., 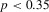). Parameters: 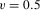. SA, sex allocation.

The gray regions in Figure 3 depict the analytically obtained condition for successful invasion by the condition-independent brood feminizer 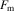. Successful invasion by 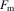 requires that sons are more costly than daughters (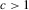), because this causes mothers to favor a more female-biased sex ratio than their offspring (see also Fig. S2). Similar to the invasion by Y, evolutionary outcomes can be separated in three different regions dependent on the frequency of the poor environment *p* (see Fig. 3). If *p* is low, we find that condition-dependent sex allocation expressed in the zygote (i.e., ESD) is replaced by condition-independent sex allocation expressed by the mother. The eventual frequency of the 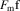 genotype results from the notion that 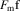 mothers produce all-female broods, half of which bear genotype 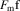 themselves, whereas the other half have genotype ff. These 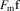 daughters continue to produce exclusively daughters themselves, whereas ff daughters produce exclusively sons, as their sex allocation is determined by the offspring's loci, which have evolved toward exclusive male development 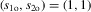 to counter the presence of the feminizer. Consequently, conflict results in a scenario where—regardless of the environment—*F*_m_f mothers produce all-female broods, whereas others produce all-male broods, which is defined as monogeny (Ullerich 1984). Moreover, note that the frequency of the 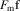 genotype is equal to the population-wide proportion of daughters being produced, achieving a frequency that is equal to the condition-independent, Fisherian sex ratio optimum 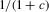.

For intermediate frequencies of the poor environment, condition-dependent sex allocation is robust to invasion and is maintained. Although sex-allocation equilibria in environment 2 diverge between mothers and offspring, 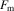 does not always invade as it leads to the maladaptive production of daughters in the poor environment 1. Only when the poor environment becomes more prevalent (*p* higher), divergence in sex-allocation equilibria between parent and offspring selects for the invasion by *F*_m_. The resulting coevolution now leads to a stable mixture of condition-independent (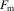) and condition-dependent 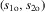 sex-determining factors, whereas the sex-allocation equilibria are shifted toward condition-dependent sex allocation expressed by the mother (cf. Fig. 3A, C).

### IMPERFECT ENVIRONMENTAL ASSESSMENT

So far, we have assumed that mothers always correctly perceive the state of the environment or their own condition. In Section S7 of the Supporting Information, we relax this assumption by allowing for errors in perception of the environment: with probability 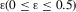, individuals perceive the current environment to be in a state that is opposite to its actual state.

**Figure 4:**
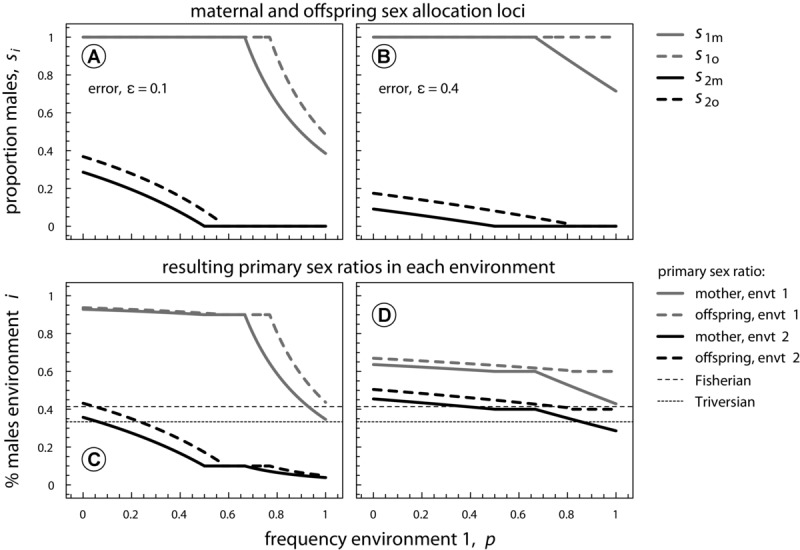
Analytical results that show how errors in environmental perception reduce condition-dependent sex-ratio biases, and increase the range of environmental frequencies *p* where parent–offspring conflict occurs. Panels A,B: maternal and offspring sex allocation strategies *s*_1_ and *s*_2_ become more extreme with an increasing environmental error probability ε. Panels C,D: unsurprisingly, with increasing error, the resulting sex ratios that are actually produced in each environment (e.g., 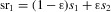) become less biased. In case environmental information is completely randomized, 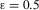, primary sex ratios will converge to Fisherian sex ratio optima 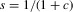 when sex-allocation loci are expressed in the mother and Triversian sex ratio optima 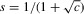 when sex-allocation loci are expressed in the zygote. Parameters: 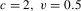.

Unsurprisingly, Figure 4 shows that nonzero errors reduce sex-ratio biases in each environment, until sex ratios for both parent and offspring finally converge toward their classical condition-independent equilibria (see eq. 2) when 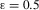. In the previous sections, we showed that whenever mothers and offspring both favor a bang-bang sex-allocation strategy 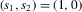, parent–offspring conflict is absent. However, since ε reduces the parameter space in which a bang-bang strategy is achieved, increased assessment errors also increase the parameter space in which parent–offspring conflict occurs (compare Fig. 4B with Fig. 1A).

#### Coevolution between perception errors and sex allocation

**Figure 5:**
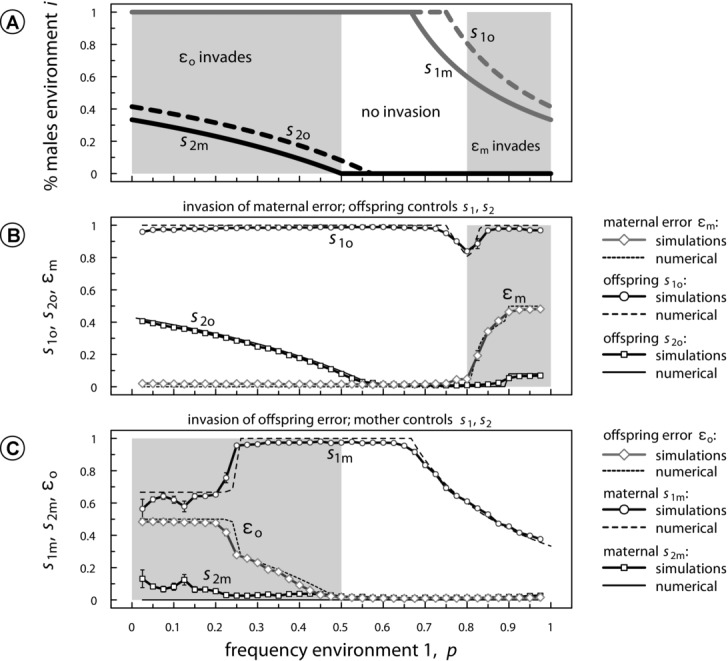
The invasion by nonzero environmental perception errors ε expressed by the mother (ε_m_) or offspring (ε_o_). Panel A: analytical results depicting when mutants with 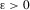 invade (gray areas). Invasion by 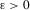 occurs whenever the desired sex-allocation optimum in one environment is closer to 0.5 than the current sex allocation in that environment. Panel B: numerical iterations and individual-based simulations depicting the invasion by a maternal factor that increases offspring perception errors 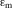 and subsequent coevolution of the offspring sex allocation loci 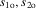. Panel C: invasion by an offspring factor that increases maternal perception errors 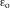 and subsequent coevolution of the maternal sex allocation loci 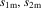. Parameters: 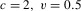.

Because sex ratios become less biased with increasing degrees of a perception error ε (Fig. 4), this also begs the question whether nonzero values of ε may be selectively favored by either parents or offspring, to achieve sex ratios closer to their respective optima. For example, parents may scramble information available to offspring by adjusting the natal environment, while liveborn offspring could secrete hormones into the maternal bloodstream (see Discussion). Figures 5 and S6 shows that conflict can indeed favor the evolution of such mechanisms that give rise to perceptual errors. Figure 5B shows, for example, that maternal induction of perception errors in offspring 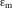 invades, whenever the good environment is relatively common. Under these conditions, mothers in environment 1 favor a sex ratio less biased from 0.5 than their offspring, and reducing the reliability of information available to offspring reduces the sex-ratio bias accordingly. However, 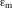 only invades whenever environment 2 is relatively scarce, so that the benefits of producing a less-biased sex ratio in environment 1 outweigh the negative effects of diverging from the sex-allocation equilibrium in environment 2. The coevolutionary outcome is either a weaker form of condition-dependent sex allocation, when perception errors evolve in the range 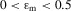, or effectively condition-independent sex allocation when 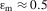.

A similar pattern is observed when mothers control sex allocation and offspring evolve a trait ε_o_ that reduces the amount of information available to mothers. We find that 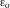 invades whenever maternal sex ratios are more biased away from equality than offspring sex ratios (see Fig. 5C). Again, 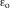 either evolves to intermediate levels, weakening condition-dependent sex allocation, or toward 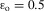, replacing condition-dependent sex allocation by condition-independent sex allocation. Although the effective replacement of condition-dependent sex allocation by condition-independent sex allocation through invading 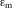 or 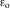 occurs only in a limited region of parameter space (see Fig. S6), it shows that genetic conflicts over sex determination can also be resolved by behavioral or hormonal factors that do not directly involve modifiers within the sex-determining cascade.

## Discussion

Although the role of genetic conflicts in the evolution of sex-determining mechanisms is increasingly appreciated (Werren and Beukeboom 1998; Burt and Trivers 2006), existing predictions mainly focus on conflicts in the context of genetic sex determination (GSD) (e.g., Rigaud and Juchault 1993; Werren et al. 2002; Van Doorn and Kirkpatrick 2007; Kozielska et al. 2009; Kuijper and Pen 2010), whereas environmental or conditional influences on sex determination have seen sparse attention in this context. The current study shows, however, that genetic conflicts may also affect the evolutionary maintenance of condition-dependent sex-determining systems, such as temperature-dependent sex allocation (Valenzuela and Lance 2004) or maternal control of sex allocation based on maternal condition (Trivers and Willard 1973) or host size (Charnov et al. 1981).

Our model suggests that conflicts between maternally expressed and zygotically expressed genes over condition-dependent sex allocation can lead to the invasion by sex factors that are independent of condition, such as sex chromosomes. However, invasion by such factors is highly contingent on the relative frequencies of both environments. For those environmental frequencies close to where both parents and offspring both favor pure sex allocation strategies, invasion is precluded (e.g., see Figs. 2, 3). This robustness against invasion occurs because any invading condition-independent sex factors will only benefit either parent or offspring in one environment, while often being selected against in the other environment. Hence, only when the former environment is much more common than the latter will invasion by the condition-independent sex factor ensue. This robustness against invasion contrasts with previous theoretical predictions regarding parent–offspring conflict over condition-independent sex allocation (Werren et al. 2002; Pen 2006), where the invasion by novel sex factors ensues whenever progeny sex ratios affect the fitness of young (e.g., by means of divergent sex-specific production costs as in the current study). If sex determination is condition-dependent, however, the existence of a divergence in sex-specific production costs does not necessarily predict successful invasion by novel sex factors.

Upon successful invasion, coevolution between the different sex factors gives rise to two possible outcomes. The first coevolutionary outcome is a replacement of condition-dependent sex determination by different forms of condition-independent sex determination. When the ancestral condition-dependent sex-determining system is expressed by the mother, it is either replaced by male heterogamety (XX-XY) when sons are more costly than daughters (Fig. 2) or female heterogamety (ZW-WW) when daughters are more costly than sons (Fig. 3) (Werren et al. 2002). Alternatively, when ancestral condition-dependent sex allocation is controlled by the offspring, conflict-driven invasion by maternal sex factors may lead to monogeny (Figs. 3, S5), where some mothers produce all-female broods, whereas others produce all-male broods regardless of the environment. Transitions such as these could potentially resemble transitions from condition-dependent sex-determining systems such as ESD to male or female heterogamety, as observed in vertebrate groups such as fish (Mank et al. 2006) or lizards (Sarre et al. 2011).

The second coevolutionary outcome that results from the invasion by condition-independent sex factors is a stable coexistence of condition-dependent and condition-independent sex factors. This outcome occurs when the invasion by a condition-independent sex factor leads to a sex ratio in one environment that is closer to either the maternal or offspring optimum, but is selectively neutral in the other environment. Such selective neutrality occurs in those environments in which the ancestral condition-dependent sex-determining system produces a pure sex ratio (i.e., either 100% sons or daughters) that matches the phenotype of the invading sex factor (masculinizer or feminizer, respectively). Consequently, the invading sex factor will only affect the sex ratios in one environment, acting effectively as a modifier of condition-dependent sex allocation that brings the sex ratio closer to either the maternal or offspring optimum. Hence, the invading sex factor is effectively integrated in the condition-dependent sex-determining cascade, while condition-dependent sex allocation is maintained (although control shifts from mother to offspring or vice versa). Our study thus suggests that parent–offspring conflict could explain observations in which sex chromosomes are stably maintained in species that have ESD as a form of condition-dependent sex allocation (Lagomarsino and Conover 1993; Shine et al. 2002; Quinn et al. 2007; Radder et al. 2008; Baroiller et al. 2009; Alho et al. 2010). Additionally, it also provides an evolutionary explanation for recent findings that both maternal and offspring factors may contribute to condition-dependent sex determination (e.g., Bowden et al. 2000; Warner et al. 2008; Radder et al. 2009, reviewed in Uller and Helanterä 2011).

Apart from the invasion by unconditional sex factors, genetic conflicts over condition-dependent sex allocation may also be resolved at the perceptual level (see Fig. 5). Our model shows that one party may evolve a perceptual error to manipulate sex-allocation decisions expressed by the other party. Currently, we can only speculate about the traits that could affect perception of the environment to either mother or offspring. A promising candidate behavior is maternal basking behavior, which has recently been associated with temperature-dependent sex determination in viviparous lizards (Wapstra et al. 2004); in the case of offspring control over condition-dependent sex determination, mothers could, for example, change their basking behavior, so that variation in temperatures experienced by the offspring is out of touch with actual temperature variation. When mothers are in control over condition-dependent sex determination, offspring may manipulate mothers by releasing hormones into the maternal bloodstream, which could putatively alter maternal perception of the environment (e.g., perception of temperature or population density). In many reptiles, embryos release a variety of hormones already early in development (Xavier et al. 1988; Guillette 1989); although most of these factors are postulated to be involved in parent–offspring conflict over maternal nutrition (Crespi and Semeniuk 2004), the actual function of these hormones is yet awaiting further exploration. Analogously, in oviparous species with offspring control over condition-dependent sex determination, mothers might influence the reliability of information available to offspring by changing the structure of the egg or the structure of the nest (e.g., Shine and Harlow 1996; Weisrock and Janzen 1999; Morjan 2003), which affects heat exchange and potentially could reduce offspring sensitivity to different temperatures. In oviparous species, however, there is a reduced scope for offspring traits that manipulate maternal perception, with expression of such traits being restricted to early developmental stages before eggshell formation prevents the release of offspring hormones. Although these aforementioned mechanisms are speculative, our model highlights that conflicts over condition-dependent sex allocation do not exclusively lead to evolutionary changes within the sex-determining cascade itself. Alongside genetic sex factors, hormones or behaviors that alter environmental information could thus potentially play an important role as well in transitions between sex-determining mechanisms.

Indications for the role of parent–offspring conflict in driving transitions from condition-dependent to condition-independent sex determination may be found in cases where both maternal and offspring sex factors contribute to sexual development, for which there is now some initial evidence (Saillant et al. 2003; Navarro-Martín et al. 2011). Specifically, our model predicts that when the upstream environment-independent sex factor is controlled by the zygote (as is likely to be the case in taxa with GSD, where male or female heterogamety is likely to have replaced ESD expressed by the mother), we predict that downstream elements in the sex-determining cascade should be controlled by the mother. Moreover, these maternally controlled downstream elements should exhibit a temperature insensitive gene expression pattern in taxa with GSD, whereas homologs of these genes in closely related taxa with ESD should be highly sensitive to temperature. Predictions such as these could be tested in the foreseeable future, given the increasing molecular knowledge about sex determination in phylogenetic groups that contain taxa where the environment (by means of temperature, condition, or population density) affects sex determination (Quinn et al. 2007 2011; Feldmeyer et al. 2008; Marshall Graves 2008; Sarre et al. 2011).

An aspect left unexplained by our model of parent–offspring conflict are transitions from condition-independent to condition-dependent sex determination, as opposed to the reverse transition studied here. The current model shows that conflict leads to maladaptive outcomes, in the sense that condition-independent sex allocation may evolve when both parents and offspring favor condition-dependent sex allocation. It is much more difficult, however, to envisage the reverse scenario in which conflict leads to maladaptive condition-dependent sex allocation, whereas condition-independent sex allocation is selectively favored by both parents and offspring. One hypothetical way through which this might occur is when invading sex modifiers disturb the molecular machinery of the existing sex-determining cascade (Frank and Crespi 2011), thereby leading to a reduction in the canalization of environmental factors (Debat and David 2001) that impinge on sex determination. Consequently, the lack of canalization may then lead to a maladaptive dependence on environment or condition. A more likely mechanism, however, for conflict-driven transitions from condition-independent toward condition-dependent sex determination is when condition-dependent sex allocation is adaptive for one party involved in the conflict, but not the other. Such scenarios may, for example, occur when phenotypic plasticity inherent in condition-dependent sex allocation entails costs (Auld et al. 2010) that accrue to the parent, but not to the offspring. For example, environmental assessment costs paid by the mother may selectively disfavor plastic sex allocation expressed by the mother, whereas offspring would selectively favor mothers to assess the environment. In general, the consequences of genetic conflicts over the cost of phenotypic plasticity deserves further attention in a broader context of life-history evolution.

Our model suggests several possibilities for future theoretical analyses. For example, the current study assumes that only the maternal breeding environment affects sex allocation, whereas in reality environmental variation across a larger time span (e.g., a mother's natal environment) should be taken into account. When sex allocation would, for example, be based on the natal environment, any mismatch between the natal and later breeding environment (e.g., due to dispersal, *p*) would reduce the benefit of condition-dependent sex allocation (see also Fischer et al. 2011; Kuijper and Johnstone 2013; Fig. 4). Consequently, parent–offspring conflict would then revert back to classical condition-independent predictions (e.g., Trivers 1974; Werren et al. 2002; Pen 2006). Note, however, that the natal environment may have a more complicated role to play, through the transmission of natal condition to offspring of a particular sex (Leimar 1996) or via cultural inheritance of the natal environment (Freedberg and Wade 2001). Consequently, future studies should assess the importance of such transgenerational effects on parent–offspring conflict over the sex ratio.

Another caveat of the current model is that it focuses on discrete environmental variation only. Previous studies have shown that continuous patterns of environmental variation lead to a larger predominance of “bang-bang” sex-allocation strategies, where the sex ratio reaction norm on the environment follows a step function (e.g., Charnov 1982; Frank 1987; Charnov and Bull 1989; Van Dooren and Leimar 2003). In other words, continuous environments selectively favor individuals that produce only males in one part of the environmental continuum, and produce only females throughout the remainder. As illustrated by the gray areas of Figure 1, the presence of bang-bang sex-allocation patterns, where both parents and offspring favor an extreme sex ratio, eliminates the scope for parent–offspring conflict. Conflict could then only persist at that part of the continuum in which sex ratios switch from exclusively male-biased to female-biased, as offspring could favor different a switch point in comparison to mothers. In general, theoretical models that predict bang-bang sex ratios in continuous environments would thus suggest that parent–offspring conflict may be less likely in continuous environments.

Empirical evidence shows, however, that bang-bang sex ratios are not necessarily the norm, with numerous studies highlighting that sex ratio reaction norms have a more gradual shape over the range of biologically relevant environments (e.g., Charnov et al. 1981; Ospina-Alvarez and Piferrer 2008; Warner and Shine 2008). Moreover, recent theoretical studies have shown that developmental noise (Van Dooren and Leimar 2003) and interactions with relatives through limited dispersal (Wild and West 2007) may drastically reduce the scope for “bang-bang” sex allocation. Such aspects should be considered in future studies that aim to study sex-ratio conflicts in more complicated continuous environments. Moreover, aspects such as interactions with relatives may also have important additional effects, as competition among kin may increase the scope for parent–offspring conflict (Werren and Hatcher 2000; Pen 2006; Kuijper and Johnstone 2012). Lastly, the current study considers only dispersal before mating, whereas dispersal after mating is not conducive to condition-dependent sex allocation (see Supporting Information). However, we have not assessed more complicated scenarios, where males are able to mate prior to dispersal and then mate again after dispersal. In conclusion, numerous opportunities thus remain to improve our understanding of role of genetic conflicts in the evolution of condition-dependent sex allocation.

## Appendix A: Maternal Control Over Sex Allocation

The matrix  (hereafter **B** represents the invasion dynamics of a mutant mother with strategy  in a resident population with sex-allocation strategy

A1

Mutant strategies appear only in the first and second columns of **B**, because we consider maternal control of the sex ratio, so any mutant sex ratio strategy will only be expressed in females. All entries in the matrix are multiplied by the constant 1/2, which considers the probability that a mutant sex ratio gene copy has a probability of  of being passed on from a parent to its offspring. Additionally, the model considers the growth rate of a mutant individual relative to the resident (Mylius and Diekmann 1995), hence we scale the matrix **B** by , where λ is the dominant eigenvalue of the resident transition matrix **A** in equation 1.

## Relative frequencies and reproductive values

For consistency, it is required that all females (having relative frequencies  and  when breeding in environments 1 and 2, respectively) have the same reproductive output as all males :

A2

where  is the *i*th column of the resident transition matrix **A**. Using this equation, we can easily find the dominant eigenvalue λ of **A**. Let  be the dominant right eigenvector of **A**, containing the stable relative class frequencies (T denotes transposition). This eigenvector is given by , or . Substituting (A2) into this equation, we get , where . Simultaneously solving for λ and *y*_f1_ and *y*_f2_ then yields

A3

and

A4

Similarly, we can solve  to obtain the elements of the dominant left eigenvector  of **A**, containing the reproductive values. These values are quite long and not particularly informative, so we will not show them here.

## Selection differentials

We calculate the selection differentials on  using equations 2 and 3 in the main text. In the results below, the positive, constant  in the denominator is not included, because it does not play a role in the calculation of equilibria. The selection differentials for the mutant sex allocation strategy are given by

A5

where . Solving both selection differentials in (A5) simultaneously requires that , which only holds in the trivial case whenever . Therefore, we focus on cases where one or both strategies are at a boundary. For a boundary strategy   to be is stable, the following should hold:

A6

In case one of both sex-allocation traits is at its boundary, but the other trait  is mixed, the following condition applies for the stability of the boundary strategy:

A7

For the mixed strategy, we assess whether evolution actually proceeds toward the mixed strategy when perturbed away from it (i.e., convergence stability; Geritz et al. 1998; Otto and Day 2007) by calculating

A8

As conditions for convergence stability are tedious for the offspring sex allocation strategies, we do not show these results here. Analytical results (maternal strategies) and numerical results (offspring) show, however, that any mixed sex allocation strategy is always convergence stable. In addition, we also assess whether the mixed strategy is evolutionarily stable by calculating

9

Numerical analyses show, however, that this term vanishes for all scenarios considered, requiring the analysis of higher order terms. Instead, we relied on individual-based simulations (which necessarily include higher order terms), where we did not find any cases where evolutionary branching occurred.

## OFFSPRING CONTROL OVER SEX ALLOCATION

The matrix  (hereafter ) represents the invasion dynamics of a mutant offspring with sex-allocation strategy  in a brood with siblings having sex-allocation strategies , while the population sex allocation strategy is given by

A10

The numbers of males and females resulting from a brood with a mutant offspring are now dependent on both the mutant and the sibling allocation strategies

Using equation 4, selection differentials for sex-allocation strategies that are expressed in the zygote are

A11

Again, stability of boundary equilibria results in three qualitatively different regions, whose boundaries are represented in Table 1.

## Supporting Information

Supporting Information S1 Timing of male dispersal. **Supporting Information S2**. Invasion by a dominant offspring masculinizer. **Supporting Information S3**. Invasion by a dominant offspring feminizer. **Supporting Information S4**. Invasion conditions of offspring sex modifiers: summary. **Supporting Information S5**. Invasion by a maternal brood masculinizer. **Supporting Information S6**. Invasion by a maternal brood feminizer. **Supporting Information S7**. Errors in environmental perception. **Figure S1**. A comparison of maternal  (thick solid lines) and offspring  (thick dashed lines) sex allocation strategies from Table 1 when varying survival of daughters in environment 1, *v* (panels A–C) and the cost of producing sons versus daughters *c* (panels D–F). **Figure S2**. Summary of the analytically obtained invasion conditions for environment-independent masculinizers (panel A) and feminizers (panel B) expressed in the zygote when condition-dependent sex allocation is controlled by the mother. **Figure S3**. Example individual-based simulation showing the successful invasion by a dominant masculinizer Y expressed in the zygote. **Figure S4**. Invasion by a dominant feminizer (*W*) expressed in the zygote, when condition-dependent sex allocation is controlled by the mother  and when daughters are twice as costly as sons (*c* = 0.5). **Figure S5**. Invasion by a dominant masculinizer *M*_m_ expressed in the mother, when condition-dependent sex allocation is controlled by the offspring ( and when daughters are twice as costly as sons (*c* = 0.5). **Figure S6**. Coevolution between perception errors ε and condition-dependent sex-allocation loci (*s*_1_, *s*_2_) resulting from numerical iterations of the analytical model.
