# Supplementary material for: Conflict over condition-dependent sex allocation can lead to mixed sex-determination systems
Source: Evolution. 2014 Oct 20;68(11):3229–47. doi: 10.1111/evo.12513 (PMC4241047; doi:10.1111/evo.12513)
Supplement: Supporting Information S1 — Timing of male dispersal. Supporting Information S2. Invasion by a dominant offspring masculinizer. Supporting Information S3. Invasion by a dominant offspring feminizer. Supporting Information S4. Invasion conditions of offspring sex modifiers: summary. Supporting Information S5. Invasion by a maternal brood masculinizer. Supporting Information S6. Invasion by a maternal brood feminizer. Supporting Information S7. Errors in environmental perception. Figure S1. A comparison of maternal (thick solid lines) and offspring (thick dashed lines) sex allocation strategies from Table 1 when varying survival of daughters in environment 1, v (panels A–C) and the cost of producing sons versus daughters c (panels D–F). Figure S2. Summary of the analytically obtained invasion conditions for environment-independent masculinizers (panel A) and feminizers (panel B) expressed in the zygote when condition-dependent sex allocation is controlled by the mother. Figure S3. Example individual-based simulation showing the successful invasion by a dominant masculinizer Y expressed in the zygote. Figure S4. Invasion by a dominant feminizer (W) expressed in the zygote, when condition-dependent sex allocation is controlled by the mother and when daughters are twice as costly as sons (c = 0.5). Figure S5. Invasion by a dominant masculinizer Mm expressed in the mother, when condition-dependent sex allocation is controlled by the offspring ( and when daughters are twice as costly as sons (c = 0.5). Figure S6. Coevolution between perception errors ε and condition-dependent sex-allocation loci (s1, s2) resulting from numerical iterations of the analytical model. [file evo0068-3229-SD1.pdf]

Conflict over condition-dependent sex allocation can lead to mixed sex-determination systems: supplementary information

Bram Kuijper & Ido Pen

S1 Timing of male dispersal

Dispersal after mating In case males disperse after mating, they become effectively philopatric: males achieve all their reproductive success in their natal environment, after which they have no effect on the population dynamics anymore. In order to incorporate male dispersal after mating, we therefore have to take into account the numbers of males that breed (and have been born) in environments 1 and 2. To model this, we assume that the population consists of four classes of individuals. First, there are n_{f1} females that breed in environment 1, n_{f2} females that breed in environment 2, n_{m1} males that are born and breed in environment 1 and n_{m2} males that are born in and breed in environment 2. Based on the transition matrix in eq. (1) of the main text, we now analyze the model $[n_{\text{f1}}, n_{\text{f2}}, n_{\text{m1}}, n_{\text{m2}}]^{\text{T}}_{t+1} = \mathbf{A}[n_{\text{f1}}, n_{\text{f2}}, n_{\text{m1}}, n_{\text{m2}}]^{\text{T}}_{t}$ (T denoting transposition), where $\mathbf{A}$ is a matrix that governs transitions between the four different classes

$$\mathbf{A} = \frac{1}{2}\begin{bmatrix} pvf_1(s_1) & pf_2(s_2) & p\frac{y_{\text{f1}}}{y_{\text{m1}}}vf_1(s_1) & p\frac{y_{\text{f2}}}{y_{\text{m2}}}f_2(s_2) \\ (1-p)vf_1(s_1) & (1-p)f_2(s_2) & (1-p)\frac{y_{\text{f1}}}{y_{\text{m1}}}vf_1(s_1) & (1-p)\frac{y_{\text{f2}}}{y_{\text{m2}}}f_2(s_2) \\ m_1(s_1) & 0 & \frac{y_{\text{f1}}}{y_{\text{m1}}}m_1(s_1) & 0 \\ 0 & m_2(s_2) & 0 & \frac{y_{\text{f2}}}{y_{\text{m2}}}m_2(s_2) \end{bmatrix}. \tag{S1}$$

For example, entry a_{31} reflects the contribution of a female breeding in a type 1 environment to a male born in a type 1 environment, who will also breed in his natal environment. Similarly, entry a_{41} shows that a female breeding in environment 1 does not contribute to the presence of males in a type 2 environment, as her sons will only mate in environment 1. Entry a_{13} reflects the contribution of a male breeding in environment 1 to females breeding in the same environment. His mating success with females born in that environment is given by the frequency of females breeding in environment 1, y_{f1}, relative to the total number of males breeding in environment 1, y_{m1}. Subsequently, this term needs to be multiplied by the survival probability v of his daughters in that environment times the sex ratio of the brood $f_1(s_1)$, times the probability p that a daughter will settle –in the next generation– in environment 1. The other entries can be derived in a similar fashion.

Solving for the right eigenvector we obtain (see eqns. A3, A4)

$$\lambda = (1-p)f_2(s_2) + pvf_1(s_1). \tag{S2}$$

and

$$\mathbf{y}^{\text{T}} = [y_{\text{f1}}, y_{\text{f2}}, y_{\text{m1}}, y_{\text{m2}}] = \left[\ p,\quad 1-p,\quad \frac{pm_1(s_1)}{pvf_1(s_1)+(1-p)f_2(s_2)},\ \frac{(1-p)m_2(s_1)}{pvf_1(s_1)+(1-p)f_2(s_2)}\right]. \tag{S3}$$

From the left eigenvector we can also derive the left eigenvector of reproductive values $\mathbf{z}$

$$\mathbf{z} = \left[\ 1,\quad \frac{\gamma_1(1-s_2)}{\gamma_2(1-s_1)v},\quad \frac{(1-p)(1-s_2)\gamma_1+p(1-s_1)\gamma_2 v}{s_1-(1-c)s_1 s_2},\quad \frac{(1-s_2)[(1-p)\gamma_1(1-s_2)+p(1-s_1)\gamma_2 v]}{(1-s_1)s_2\gamma_2 v}\ \right]. \tag{S4}$$

From $\mathbf{A}$, we can then derive the mutant transition matrices $\mathbf{B}_{\text{mother}}$ and $\mathbf{B}_{\text{offspring}}$ when sex allocation is expressed in the mother and offspring respectively:

$$\mathbf{B}_{\text{mother}} = \frac{1}{2\lambda}\begin{bmatrix} pvf_1(\hat{s}_1) & pf_2(\hat{s}_2) & p\frac{y_{\text{f1}}}{y_{\text{m1}}}vf_1(s_1) & p\frac{y_{\text{f2}}}{y_{\text{m2}}}f_2(s_2) \\ (1-p)vf_1(\hat{s}_1) & (1-p)f_2(\hat{s}_2) & (1-p)\frac{y_{\text{f1}}}{y_{\text{m1}}}vf_1(s_1) & (1-p)\frac{y_{\text{f2}}}{y_{\text{m2}}}f_2(s_2) \\ m_1(\hat{s}_1) & 0 & \frac{y_{\text{f1}}}{y_{\text{m1}}}m_1(s_1) & 0 \\ 0 & m_2(\hat{s}_2) & 0 & \frac{y_{\text{f2}}}{y_{\text{m2}}}m_2(s_2) \end{bmatrix} \tag{S5}$$

$$\mathbf{B}_{\text{offspring}} = \frac{1}{2\lambda}\begin{bmatrix} pvf_1(\hat{s}_1,\tilde{s}_1) & pf_2(\hat{s}_2,\tilde{s}_2) & p\frac{y_{\text{f1}}}{y_{\text{m1}}}vf_1(\hat{s}_1,\tilde{s}_1) & p\frac{y_{\text{f2}}}{y_{\text{m2}}}f_2(\hat{s}_2,\tilde{s}_2) \\ (1-p)vf_1(\hat{s}_1,\tilde{s}_1) & (1-p)f_2(\hat{s}_2,\tilde{s}_2) & (1-p)\frac{y_{\text{f1}}}{y_{\text{m1}}}vf_1(\hat{s}_1,\tilde{s}_1) & (1-p)\frac{y_{\text{f2}}}{y_{\text{m2}}}f_2(\hat{s}_2,\tilde{s}_2) \\ m_1(\hat{s}_1,\tilde{s}_1) & 0 & \frac{y_{\text{f1}}}{y_{\text{m1}}}m_1(\hat{s}_1,\tilde{s}_1) & 0 \\ 0 & m_2(\hat{s}_2,\tilde{s}_2) & 0 & \frac{y_{\text{f2}}}{y_{\text{m2}}}m_2(\hat{s}_2,\tilde{s}_2) \end{bmatrix} \tag{S6}$$

and calculate selection gradients for the sex allocation strategy $(s_{1\text{m}}, s_{2\text{m}})$ expressed by mothers according to eq. (3) in the main text. This yields selection gradients of an identical form as in eq. (A5)

$$\begin{aligned} \left.\frac{\mathrm{d}W}{\mathrm{d}\hat{s}_1}\right|_{\substack{\hat{s}_1=s_1\\ \hat{s}_2=s_2}} &= \frac{y_{\text{f1}}}{2\lambda\gamma_1^2}\left[z_{\text{m1}} - cv(z_{\text{f1}}p + z_{\text{f2}}(1-p))\right] \\ \left.\frac{\mathrm{d}W}{\mathrm{d}\hat{s}_2}\right|_{\substack{\hat{s}_1=s_1\\ \hat{s}_2=s_2}} &= \frac{y_{\text{f2}}}{2\lambda\gamma_2^2}\left[z_{\text{m2}} - c(z_{\text{f1}}p + z_{\text{f2}}(1-p))\right], \end{aligned} \tag{S7}$$

yet when substituting for $\mathbf{y}$ and $\mathbf{z}$ we obtain

$$s_{1\text{m}}^{\star} = \frac{1}{1+c}, s_{2\text{m}}^{\star} = \frac{1}{1+c} \tag{S8}$$

Similarly for the sex allocation strategy $(s_{1\text{o}}, s_{2\text{o}})$ expressed by offspring (see eq. [4] in the main text), we obtain

$$\begin{aligned} \left.\frac{\mathrm{d}W}{\mathrm{d}\hat{s}_1}\right|_{\substack{\hat{s}_1=\tilde{s}_1=s_1\\ \hat{s}_2=\tilde{s}_2=s_2}} &= \frac{y_{\text{f1}}}{\gamma_1\lambda}\left(z_{\text{m1}} - v(z_{\text{f1}}p + z_{\text{f2}}(1-p)) + \frac{1}{2\gamma_1}(1-c)\left[z_{\text{m1}}s_1 - v(1-s_1)(z_{\text{f1}}p + z_{\text{f2}}(1-p))\right]\right), \\ \left.\frac{\mathrm{d}W}{\mathrm{d}\hat{s}_2}\right|_{\substack{\hat{s}_1=\tilde{s}_1=s_1\\ \hat{s}_2=\tilde{s}_2=s_2}} &= \frac{y_{\text{f2}}}{\gamma_2\lambda}\left(z_{\text{m2}} - (z_{\text{f1}}p + z_{\text{f2}}(1-p)) + \frac{1}{2\gamma_2}(1-c)\left[z_{\text{m2}}s_2 - (1-s_2)(z_{\text{f1}}p + z_{\text{f2}}(1-p))\right]\right). \end{aligned} \tag{S9}$$

After some algebra, solving for these selection gradients yields

$$s_{1\text{o}}^{\star} = \frac{1}{1+\sqrt{c}}, s_{2\text{o}}^{\star} = \frac{1}{1+\sqrt{c}} \tag{S10}$$

so that condition-dependent sex allocation is necessarily absent in case of male philopatry for both parents and offspring.

Dispersal before mating In case of dispersal before mating, we assume that males disperse to environment 1 with probability d_1, whereas they disperse to environment 2 with probability $1-d_1$, where they mate. Again, the population consists of four classes of individuals. First, there are n_{f1} females that breed in environment 1, n_{f2} females that breed in environment 2, n_{m1} males that breed in environment 1 and n_{m2} males that breed in environment 2. We then obtain the following resident transition matrix

$$\mathbf{A} = \frac{1}{2}\begin{bmatrix} pvf_1(s_1) & pf_2(s_2) & p\frac{y_{\text{f1}}}{y_{\text{m1}}}vf_1(s_1) & p\frac{y_{\text{f2}}}{y_{\text{m2}}}f_2(s_2) \\ (1-p)vf_1(s_1) & (1-p)f_2(s_2) & (1-p)\frac{y_{\text{f1}}}{y_{\text{m1}}}vf_1(s_1) & (1-p)\frac{y_{\text{f2}}}{y_{\text{m2}}}f_2(s_2) \\ d_1m_1(s_1) & d_1m_2(s_2) & d_1\frac{y_{\text{f1}}}{y_{\text{m1}}}m_1(s_1) & d_1\frac{y_{\text{f2}}}{y_{\text{m2}}}m_2(s_2) \\ (1-d_1)m_1(s_1) & (1-d_1)m_2(s_2) & (1-d_1)\frac{y_{\text{f1}}}{y_{\text{m1}}}m_1(s_1) & (1-d_1)\frac{y_{\text{f2}}}{y_{\text{m2}}}m_2(s_2) \end{bmatrix}. \tag{S11}$$

and we have the following mutant transition matrices when (s_1, s_2) are controlled by mother and offspring respectively

$$\mathbf{B}_{\text{mother}} = \frac{1}{2\lambda}\begin{bmatrix} pvf_1(\hat{s}_1) & pf_2(\hat{s}_2) & p\frac{y_{\text{f1}}}{y_{\text{m1}}}vf_1(s_1) & p\frac{y_{\text{f2}}}{y_{\text{m2}}}f_2(s_2) \\ (1-p)vf_1(\hat{s}_1) & (1-p)f_2(\hat{s}_2) & (1-p)\frac{y_{\text{f1}}}{y_{\text{m1}}}vf_1(s_1) & (1-p)\frac{y_{\text{f2}}}{y_{\text{m2}}}f_2(s_2) \\ d_1m_1(\hat{s}_1) & d_1m_2(\hat{s}_2) & d_1\frac{y_{\text{f1}}}{y_{\text{m1}}}m_1(s_1) & d_1\frac{y_{\text{f2}}}{y_{\text{m2}}}m_2(s_2) \\ (1-d_1)m_1(\hat{s}_1) & (1-d_1)m_2(\hat{s}_2) & (1-d_1)\frac{y_{\text{f1}}}{y_{\text{m1}}}m_1(s_1) & (1-d_1)\frac{y_{\text{f2}}}{y_{\text{m2}}}m_2(s_2) \end{bmatrix} \tag{S12}$$

$$\mathbf{B}_{\text{offspring}} = \frac{1}{2\lambda}\begin{bmatrix} pvf_1(\hat{s}_1,\tilde{s}_1) & pf_2(\hat{s}_2,\tilde{s}_2) & p\frac{y_{\text{f1}}}{y_{\text{m1}}}vf_1(\hat{s}_1,\tilde{s}_1) & p\frac{y_{\text{f2}}}{y_{\text{m2}}}f_2(\hat{s}_2,\tilde{s}_2) \\ (1-p)vf_1(\hat{s}_1,\tilde{s}_1) & (1-p)f_2(\hat{s}_2,\tilde{s}_2) & (1-p)\frac{y_{\text{f1}}}{y_{\text{m1}}}vf_1(\hat{s}_1,\tilde{s}_1) & (1-p)\frac{y_{\text{f2}}}{y_{\text{m2}}}f_2(\hat{s}_2,\tilde{s}_2) \\ d_1m_1(\hat{s}_1,\tilde{s}_1) & d_1m_2(\hat{s}_2,\tilde{s}_2) & d_1\frac{y_{\text{f1}}}{y_{\text{m1}}}m_1(\hat{s}_1,\tilde{s}_1) & d_1\frac{y_{\text{f2}}}{y_{\text{m2}}}m_2(\hat{s}_2,\tilde{s}_2) \\ (1-d_1)m_1(\hat{s}_1,\tilde{s}_1) & (1-d_1)m_2(\hat{s}_2,\tilde{s}_2) & (1-d_1)\frac{y_{\text{f1}}}{y_{\text{m1}}}m_1(\hat{s}_1,\tilde{s}_1) & (1-d_1)\frac{y_{\text{f2}}}{y_{\text{m2}}}m_2(\hat{s}_2,\tilde{s}_2) \end{bmatrix}. \tag{S13}$$

Again, we can derive reproductive values and class frequencies as in eqns. A3, A4, as well as the selection gradient when sex allocation is expressed in the mother or zygote. After some algebra, we find the same condition-dependent sex allocation optima as in Table 1 in the main text.

S2 Invasion by a dominant offspring masculinizer

Here we consider the coevolution of a dominant offspring masculinizer and condition-dependent sex allocation (s_1, s_2) expressed by the mother. Consider three unlinked loci: the first two loci S_1 and S_2 codes for maternally controlled condition-dependent sex allocation (s_1, s_2). We assume additive gene action at these loci and assume that any gene interactions with the other locus are absent. Since under additive gene action a locus with diploid inheritance is functioning like a haploid locus, we assume (for sake of simplicity) that the S_1 and S_2 loci obey haploid inheritance (individual-based simulations asssuming diploid loci reach identical results). In the section "Maternal coevolutionary response" below, we consider the evolution at S_1 and S_2 through the successive invasion and substitution of maternal sex allocation mutants of small effect.

The third locus, Y, is expressed in the zygote; individuals having the yy genotype defer control over sex allocation to the mother, and thus become male with probability s_i or $\hat{s}_i$ dependent on whether it is born from a resident or mutant mother. In contrast, individuals having the mutant Yy genotype always develop as males, independent of their maternal genotype and the environment. While males always survive to become breeding adults, females survive with probability v in environment 1, and always survive in environment 2. Since Yy males will only mate with yy females, the genotype YY does not exist. Let the column vector $\mathbf{x}_t = [x_{1,t}, x_{2,t}, x_{3,t}, x_{4,t}]^{\mathrm{T}}$ describe the frequencies of the different types in the population at time t: $x_{1,t}$ and $x_{2,t}$ describe the frequencies of yy females breeding in patch types 1 and 2 respectively, whereas $x_{3,t}$ and $x_{4,t}$ describe the frequencies of yy males and Yy males across all patches. As the offspring masculinizer invades, selection may favor mutant mothers with slightly divergent sex allocation strategies $(\hat{s}_1, \hat{s}_2)$. Consequently, the coevolution of Y and $(\hat{s}_1, \hat{s}_2)$ leads to changes in the frequency vector $\mathbf{x}$, which is given by the dynamic $\mathbf{x}_{t+1} = \mathbf{B}\mathbf{x}_t$, where $\mathbf{B}$ is the 4×4 transition matrix

$$\mathbf{B} = \frac{1}{2\lambda}\begin{bmatrix} pv(1-\hat{s}_1)\left[\ell_{yy}h_{yy}(\hat{s}_1)+\frac{1}{2}\ell_{Yy}h_{Yy}(\hat{s}_1)\right] & p(1-\hat{s}_2)\left[\ell_{yy}h_{yy}(\hat{s}_2)+\frac{1}{2}\ell_{Yy}h_{Yy}(\hat{s}_2)\right] & p\left[q_1vh_{yy}(s_1)(1-s_1)+q_2h_{yy}(s_2)(1-s_2)\right] & \frac{1}{2}p\left[q_1vh_{Yy}(s_1)(1-s_1)+q_2h_{Yy}(s_2)(1-s_2)\right] \\ (1-p)v(1-\hat{s}_1)\left[\ell_{yy}h_{yy}(\hat{s}_1)+\frac{1}{2}\ell_{Yy}h_{Yy}(\hat{s}_1)\right] & (1-p)(1-\hat{s}_2)\left[\ell_{yy}h_{yy}(\hat{s}_2)+\frac{1}{2}\ell_{Yy}h_{Yy}(\hat{s}_2)\right] & (1-p)\left[q_1vh_{yy}(s_1)(1-s_1)+q_2h_{yy}(s_2)(1-s_2)\right] & \frac{1}{2}(1-p)\left[q_1vh_{Yy}(s_1)(1-s_1)+q_2h_{Yy}(s_2)(1-s_2)\right] \\ \ell_{yy}h_{yy}(\hat{s}_1)\hat{s}_1+\frac{1}{2}\ell_{Yy}h_{Yy}(\hat{s}_1)\hat{s}_1 & \ell_{yy}h_{yy}(\hat{s}_2)\hat{s}_2+\frac{1}{2}\ell_{Yy}h_{Yy}(\hat{s}_2)\hat{s}_2 & q_1h_{yy}(s_1)s_1+q_2h_{yy}(s_2)s_2 & \frac{1}{2}q_1h_{Yy}(s_1)s_1+\frac{1}{2}q_2h_{Yy}(s_2)s_2 \\ \frac{1}{2}\ell_{Yy}h_{Yy}(\hat{s}_1) & \frac{1}{2}\ell_{Yy}h_{Yy}(\hat{s}_2) & 0 & \frac{1}{2}q_1h_{Yy}(s_1)+\frac{1}{2}q_2h_{Yy}(s_2) \end{bmatrix}, \tag{S14}$$

where λ is the dominant eigenvalue of the resident transition matrix $\mathbf{A} \equiv \mathbf{B}|_{s_1=\hat{s}_1, s_2=\hat{s}_2}$. Additionally, $h_{yy}(s_i)$ and $h_{Yy}(s_i)$ are the fecundities of mothers mated to yy and Yy fathers respectively:

$$h_{yy}(s_i) = \frac{1}{s_i c + 1 - s_i}, \quad h_{Yy}(s_j) = \frac{1}{\frac{1}{2}(1+s_i)c + \frac{1}{2}(1-s_i)},$$

while $\ell_{yy} = x_3/(x_3+x_4)$ and $\ell_{Yy} = x_4/(x_3+x_4)$ are the probabilities that a given female mates with a yy and Yy male (assuming random mating). Lastly, $q_1 = x_1/(x_3+x_4)$ and $q_2 = x_2/(x_3+x_4)$ are the probabilities that a given male successfully achieves a mating with a female that breeds in environment 1 or in patch 2 respectively.

To briefly illustrate the model in eq. (S14), let us illustrate two entries of the matrix $\mathbf{B}$: entry b_{11} describes the proportion of yy daughters breeding in environment 1 produced by a yy mother with mutant sex allocation strategy $(\hat{s}_1, \hat{s}_2)$, who breeds herself in environment 1 too. With probability ℓ_{yy}, the mother has mated with an yy male. Subsequently, her fecundity is $h_{yy}(\hat{s}_1)$, and a proportion $1-\hat{s}_1$ of these offspring develop as daughters. Alternatively, with probability ℓ_{Yy}, she mates with a Yy male, so that her fecundity is $h_{Yy}(\hat{s}_1)$. In the latter case, half of her offspring receive the Yy genotype, and those always develop as males. The remaining half, however, receive the yy genotype, and a proportion $1-\hat{s}_1$ of them develop as daughters. Lastly, of all daughters produced in environment 1, only a proportion v will survive. Of all surviving daughters, a proportion p will breed in environment 1 in the next generation.

Entry b_{41} describes the production of Yy sons by a yy female breeding in environment 1. Such sons can only be produced when she mates with a Yy male, given by probability ℓ_{Yy}. She then has fecundity $h_{Yy}(\hat{s}_1)$ and half of all offspring receive the Yy genotype, who subsequently all develop as male

S2.1 Invasion dynamics of the masculinizer

The change in frequency of the offspring masculinizer is given by

$$x_{4,t+1} = x_{4,t}\frac{w_4}{\bar{w}} = x_{4,t}\frac{\sum_{j=1}^{4} a_{4j}x_{j,t}}{\sum_{i=1}^{4}\sum_{j=1}^{4} x_{i,t}a_{ij}x_{j,t}}, \tag{S15}$$

where a_{ij} are entries of the resident transition matrix $\mathbf{A}$. We assume that a rare masculinizer invades in a population that has reached an equilibrium maternal sex allocation strategy (s_1^*, s_2^*) given by Table 1. Conditions for invasion by a rare masculinizer can be obtained by calculating the Jacobian matrix $\mathbf{J}$ from the system $\mathbf{A}\mathbf{x}_{t=0}$, where $\mathbf{x}_{t=0} = [x_1, x_2, x_3, 0]^{\mathrm{T}}$ being the right eigenvector of $\mathbf{A}$ for the sex allocation strategy (s_1^*, s_2^*) in absence of the masculinizer. Results for $\mathbf{J}$ are tedious and not very informative, so we summarize results graphically in Supplementary Figure S2A, and the shadings in the relevant Figures in the main text.

S2.2 Maternal coevolutionary response

Upon invasion by the masculinizer, we iterate the dynamic in eq. (S15) and update the values of the eigenvector $\mathbf{z}$ accordingly during each generation. When the masculinizer achieves its equilibrium frequency x_4^*, we then consider a coevolutionary response by mutant mothers, which corresponds to viewing the evolutionary process as a gradual, mutation-limited process (refs). Specifically, the selection differential that describes the successful invasion by a slightly deviant sex allocation strategy $\hat{s}_i$ is given by

$$\frac{\mathrm{d}W}{\mathrm{d}\hat{s}_i} = \frac{\partial W}{\partial \hat{s}_i} = \mathbf{z}^{\mathrm{T}}\frac{\partial \mathbf{B}}{\partial \hat{s}_i}\bigg|_{\hat{s}_1=s_1,\hat{s}_2=s_2} \mathbf{x}/\mathbf{z}^{\mathrm{T}}\mathbf{x}. \tag{S16}$$

We assume that mutations in s_1 occur independently of those in s_2. Upon a successful invasion by a novel $\hat{s}_i$ mutant, it becomes the new resident sex allocation strategy $s_1 = \hat{s}_1$. Subsequently, we then update the equilibrium value of the masculinizer frequency x_4, by iterating eq. (S15) until a new equilibrium is reached. Subsequently, we repeat these steps by allowing a novel $\hat{s}_i$ mutant to invade. This process then continues until both (s_1, s_2) and x_4 achieve their equilibria.

S3 Invasion by a dominant offspring feminizer

Again, we have the dynamic $\mathbf{x}_{t+1} = \mathbf{B}\mathbf{x}_t$. Individuals carrying the ww genotype develop according to the condition-dependent sex allocation strategy (s_1, s_2) at locus A. By contrast, individuals bearing the Ww genotype always develop as females. The column $\mathbf{x} = [x_1, \ldots, x_5]^T$ describes the frequencies of the relevant genotypes in both environments, with x_1 and x_2 describe the frequencies of ww and Ww females respectively, who breed in environment 1. x_3 and x_4 are the equivalent frequencies of ww and Ww females breeding in environment 2. Lastly, x_5 reflects the frequency of males, which necessarily bear the ww genotype. The matrix $\mathbf{B}$ is thus a 5×5 transition matrix

$$\mathbf{B} = \tfrac{1}{2\lambda}\left[\begin{matrix} pvh_{ww}(\hat{s}_1)(1-\hat{s}_1) & \tfrac{1}{2}pvh_{Ww}(\hat{s}_1)(1-\hat{s}_1) & ph_{ww}(\hat{s}_2)(1-\hat{s}_2) & \tfrac{1}{2}ph_{Ww}(\hat{s}_2)(1-\hat{s}_2) \\ 0 & \tfrac{1}{2}pvh_{Ww}(\hat{s}_1) & 0 & \tfrac{1}{2}ph_{Ww}(\hat{s}_2) \\ (1-p)vh_{ww}(\hat{s}_1)(1-\hat{s}_1) & \tfrac{1}{2}(1-p)vh_{Ww}(\hat{s}_1)(1-\hat{s}_1) & (1-p)h_{ww}(\hat{s}_2)(1-\hat{s}_2) & \tfrac{1}{2}(1-p)h_{Ww}(\hat{s}_2)(1-\hat{s}_2) \\ 0 & \tfrac{1}{2}(1-p)vh_{Ww}(\hat{s}_1) & 0 & \tfrac{1}{2}(1-p)h_{Ww}(\hat{s}_2) \\ h_{ww}(\hat{s}_1)\hat{s}_1 & \tfrac{1}{2}h_{Ww}(\hat{s}_1)\hat{s}_1 & h_{ww}(\hat{s}_2)\hat{s}_2 & \tfrac{1}{2}h_{Ww}(\hat{s}_2)\hat{s}_2 \end{matrix}\right.$$

$$\left.\begin{matrix} p\left[\frac{x_1}{x_5}h_{ww}(s_1)(1-s_1)v + \frac{1}{2}\frac{x_2}{x_5}h_{Ww}(s_1)(1-s_1)v + \frac{x_3}{x_5}h_{ww}(s_2)(1-s_2) + \frac{1}{2}\frac{x_4}{x_5}h_{Ww}(s_2)(1-s_2)\right] \\ p\left[\frac{1}{2}\frac{x_2}{x_5}h_{Ww}(s_1)v + \frac{1}{2}\frac{x_4}{x_5}h_{Ww}(s_2)\right] \\ (1-p)\left[\frac{x_1}{x_5}h_{ww}(s_1)(1-s_1)v + \frac{1}{2}\frac{x_2}{x_5}h_{Ww}(s_1)(1-s_1)v + \frac{x_3}{x_5}h_{ww}(s_2)(1-s_2) + \frac{1}{2}\frac{x_4}{x_5}h_{Ww}(s_2)(1-s_2)\right] \\ (1-p)\left[\frac{1}{2}\frac{x_2}{x_5}h_{Ww}(s_1)v + \frac{1}{2}\frac{x_4}{x_5}h_{Ww}(s_2)\right] \\ \frac{x_1}{x_5}h_{ww}(s_1)s_1 + \frac{1}{2}\frac{x_2}{x_5}h_{Ww}(s_1)s_1 + \frac{x_3}{x_5}h_{ww}(s_2)s_2 + \frac{1}{2}\frac{x_4}{x_5}h_{Ww}(s_2)s_2 \end{matrix}\right]. \tag{S17}$$

Fecundities $h_{ww}(s_i)$ and $h_{Ww}(s_i)$ of mothers bearing the ww or Ww alleles respectively are given by

$$h_{ww}(s_i) = \frac{1}{s_i c + 1 - s_i}, \quad h_{Ww}(s_i) = \frac{1}{\frac{1}{2}(2 - s_i) + \frac{1}{2} s_i c}.$$

The recursion of the frequency of the offspring feminizer genotype $f(Ww)_{t+1}$ is then given by

$$f(Ww)_{t+1} = x_2 \frac{\sum_{j=1}^{5} a_{2j} x_j}{\sum_{i=1}^{5} \sum_{j=1}^{5} x_i a_{ij} x_j} + x_4 \frac{\sum_{j=1}^{5} a_{4j} x_j}{\sum_{i=1}^{5} \sum_{j=1}^{5} x_i a_{ij} x_j}, \qquad \text{(S18)}$$

where a_{ij} reflects the ijth entry of the resident version $\mathbf{A} = \mathbf{B}|_{s_1=\hat{s}_1, s_2=\hat{s}_2}$ of the transition matrix in (S17).

The coevolutionary dynamics between the feminizer expressed in the offspring and the maternally expressed condition-dependent sex allocation strategy are described by equivalent equations as for the masculinizer in eqns. (S15,S16). Invasion conditions for the feminizer expressed by offspring are given in Figure Supplementary Figure S2B.

S4 Invasion conditions of offspring sex modifiers: summary

Figure S2 depicts the invasion conditions of dominant offspring masculinizers and feminizers. Unsurprisingly, the invasion by a masculinizer is impossible when females are more costly to produce than males ($c < 1$), since offspring than prefer a more female-biased sex ratio than their mothers, so that any offspring masculinizer cannot invade (the reverse applies to offspring feminizers, which cannot invade for values $c > 1$, Figure S2B). For values of $c > 1$, the invasion by offspring masculinizers is dependent on the frequency p of the poor environment and the survival probability v of females in the poor environment. Specifically, for values of $p < 1/2$ (region I in Figure 1), we find that any masculinizer can invade regardless of the value of v (Figure S2A). For higher frequencies p of the poor environment, however, we find that the invasion by a masculinizer becomes more difficult for lower values of v. This is because lower values of v select for larger number of males to be produced in the more prevalent, poor environment. Consequently, the resulting male-biased sex ratio reduces the scope for invasion by any masculinizer. Regarding the invasion by the feminizer, we also find that higher survival v of females in the poor environment is more conducive to invasion than lower values (Figure S2B), simply because lower female survival directly reduces the invasion prospects of any feminizing allele.

S5 Invasion by a maternal brood masculinizer

Mothers carrying the *mm* genotype produce offspring which determine their own sex according to the sex allocation strategy (s_1, s_2). By contrast, heterozygous mothers bearing the $M_\mathrm{m}m$ genotype override the condition-dependent sex allocation strategy expressed by the offspring and produce only sons. The column vector $\mathbf{x} = [x_1, \ldots, x_6]^\mathrm{T}$ describes the frequencies of the different types of individuals, with x_1 and x_2 describing the frequencies of *mm* females breeding either in environment 1 or 2. Next, x_3 describes the frequency of females carrying the $M_\mathrm{m}m$ allele, breeding in either environment (the environment is irrelevant, as $M_\mathrm{m}m$ mothers produce only sons, who have identical survival prospects in either environment as assumed by our model). Lastly, x_4, x_5 and x_6 reflect the frequencies of males that either bear genotypes *mm*, $M_\mathrm{m}m$ or $M_\mathrm{m}M_\mathrm{m}$. Note that $M_\mathrm{m}M_\mathrm{m}$ females don't exist, as $M_\mathrm{m}m$ females only transmit their M_m allele to sons. The 6×6 transition matrix $\mathbf{B}$ is then given by

$$\mathbf{B} = \frac{1}{2\lambda}\begin{bmatrix} pv\left[\ell_4+\frac{1}{2}\ell_5\right]g_{mm}(\tilde{s}_1)(1-\hat{s}_1) & pv\left[\ell_4+\frac{1}{2}\ell_5\right]g_{mm}(\tilde{s}_2)(1-\hat{s}_2) & 0 & p\left[vq_1g_{mm}(\tilde{s}_1)(1-\hat{s}_1)+q_2g_{mm}(\tilde{s}_2)(1-\hat{s}_2)\right] & \frac{1}{2}p\left[vq_1g_{mm}(\tilde{s}_1)(1-\hat{s}_1)+q_2g_{mm}(\tilde{s}_2)(1-\hat{s}_2)\right] & 0 \\ (1-p)v\left[\ell_4+\frac{1}{2}\ell_5\right]g_{mm}(\tilde{s}_1)(1-\hat{s}_1) & (1-p)v\left[\ell_4+\frac{1}{2}\ell_5\right]g_{mm}(\tilde{s}_2)(1-\hat{s}_2) & 0 & (1-p)\left[vq_1g_{mm}(\tilde{s}_1)(1-\hat{s}_1)+q_2g_{mm}(\tilde{s}_2)(1-\hat{s}_2)\right] & \frac{1}{2}(1-p)\left[vq_1g_{mm}(\tilde{s}_1)(1-\hat{s}_1)+q_2g_{mm}(\tilde{s}_2)(1-\hat{s}_2)\right] & 0 \\ v\left[\frac{1}{2}\ell_5+\ell_6\right]g_{mm}(\tilde{s}_1)(1-\hat{s}_1) & v\left[\frac{1}{2}\ell_5+\ell_6\right]g_{mm}(\tilde{s}_2)(1-\hat{s}_2) & 0 & 0 & \frac{1}{2}\left[vq_1g_{mm}(\tilde{s}_1)(1-\hat{s}_1)+q_2g_{mm}(\tilde{s}_2)(1-\hat{s}_2)\right] & vq_1g_{mm}(\tilde{s}_1)(1-\hat{s}_1)+q_2g_{mm}(\tilde{s}_2)(1-\hat{s}_2) \\ \left[\ell_4+\frac{1}{2}\ell_5\right]g_{mm}(\tilde{s}_1)\hat{s}_1 & \left[\ell_4+\frac{1}{2}\ell_5\right]g_{mm}(\tilde{s}_2)\hat{s}_2 & \left[\frac{1}{2}\ell_4+\frac{1}{4}\ell_5\right]g_{M_\mathrm{m}m} & q_1g_{mm}(\tilde{s}_1)\hat{s}_1+q_2g_{mm}(\tilde{s}_2)\hat{s}_2+\frac{1}{2}q_3g_{M_\mathrm{m}m} & \frac{1}{2}\left[q_1g_{mm}(\tilde{s}_1)\hat{s}_1+q_2g_{mm}(\tilde{s}_2)\hat{s}_2\right]+\frac{1}{4}q_3g_{M_\mathrm{m}m} & 0 \\ \left[\frac{1}{2}\ell_5+\ell_6\right]g_{mm}(\tilde{s}_1)\hat{s}_1 & \left[\frac{1}{2}\ell_5+\ell_6\right]g_{mm}(\tilde{s}_2)\hat{s}_2 & \left[\frac{1}{2}\ell_4+\frac{1}{2}\ell_5+\frac{1}{2}\ell_6\right]g_{M_\mathrm{m}m} & \frac{1}{2}q_3g_{M_\mathrm{m}m} & \frac{1}{2}\left[q_1g_{mm}(\tilde{s}_1)\hat{s}_1+q_2g_{mm}(\tilde{s}_2)\hat{s}_2\right]+\frac{1}{2}q_3g_{M_\mathrm{m}m} & q_1g_{mm}(\tilde{s}_1)\hat{s}_1+q_2g_{mm}(\tilde{s}_2)\hat{s}_2+\frac{1}{2}q_3g_{M_\mathrm{m}m} \\ 0 & 0 & \left[\frac{1}{4}\ell_5+\frac{1}{2}\ell_6\right]g_{M_\mathrm{m}m} & 0 & \frac{1}{4}q_3g_{M_\mathrm{m}m} & \frac{1}{2}q_3g_{M_\mathrm{m}m} \end{bmatrix}, \tag{S19}$$

where $g_{mm}(\tilde{s}_i)$ and $g_{M_\mathrm{m}m}$ are the fecundities of mothers that bear the *mm* or $M_\mathrm{m}m$ genotypes respectively. Note that $g_{mm}(\tilde{s}_i)$ is a function of the average sex allocation strategy by the brood $\tilde{s}_i$. By contrast, $g_{M_\mathrm{m}m}$ is independent of any condition-dependent sex allocation, as all individuals develop as males:

$$g_{mm}(\tilde{s}_i) = \frac{1}{\tilde{s}_i c + 1 - \tilde{s}_i}, \quad g_{M_\mathrm{m}m} = \frac{1}{c}.$$

Next, $\ell_k = x_k/(x_4 + x_5 + x_6)$ with $k \in \{4, 5, 6\}$ describes the probability that a female mates with a *mm*, $M_\mathrm{m}m$ or $M_\mathrm{m}M_\mathrm{m}$ male. Similarly, $q_j = x_j/(x_4 + x_5 + x_6)$ with $j \in \{1, 2, 3\}$ describes the probability that a male successfully competes for a mating with *mm* females living in environment 1 or two (x_1, x_2) or an $M_\mathrm{m}m$ female (x_3).

As an example, entry b_{42} describes the number of *mm* sons produced by a *mm*-mother living in a type 2 environment. With probability ℓ_4 the *mm* mother has mated with a *mm* male, producing only *mm* offspring. With probability ℓ_5, this *mm*-mother mates with a $M_\mathrm{m}m$ male, so that half of all offspring bear the *mm* genotype. As the mother herself bears the *mm* genotype, she produces a brood of size $g_{ff}(\tilde{s}_2)$ in both cases. Individual offspring sex allocation mutants subsequently develop as males with probability $\hat{s}_2$.

The change in frequency of the maternal brood masculinizer allele is then given by

$$\begin{aligned} f(M_\mathrm{m})_t = {} & x_3 \frac{\sum_{j=1}^{6} a_{3j}x_j}{\sum_{i=1}^{6}\sum_{j=1}^{6} x_i a_{ij} x_j} + x_5 \frac{\sum_{j=1}^{6} a_{5j}x_j}{\sum_{i=1}^{6}\sum_{j=1}^{6} x_i a_{ij} x_j} \\ & + 2x_6 \frac{\sum_{j=1}^{6} a_{6j}x_j}{\sum_{i=1}^{6}\sum_{j=1}^{6} x_i a_{ij} x_j}, \end{aligned} \tag{S20}$$

where a_{ij} reflect entries from the resident transition matrix $\mathbf{A} = \mathbf{B}|_{s_1=\tilde{s}_1=\hat{s}_1, s_2=\tilde{s}_2=\hat{s}_2}$. The coevolutionary response by the offspring condition-dependent sex allocation trait s_j is given by

$$\frac{\mathrm{d}W}{\mathrm{d}\hat{s}_j} = \left.\frac{\partial W}{\partial \hat{s}_j}\right|_{\hat{s}_j=\tilde{s}_j=s_j} + R\left.\frac{\partial W}{\partial \tilde{s}_j}\right|_{\hat{s}_j=\tilde{s}_j=s_j}, \tag{S21}$$

where

$$\left.\frac{\partial W}{\partial \hat{s}_x}\right|_{\hat{s}_x=s_x} = \mathbf{z}^{\mathrm{T}} \left.\frac{\partial \mathbf{B}}{\partial \hat{s}_x}\right|_{\hat{s}_x=s_x} \mathbf{x}/\mathbf{z}^{\mathrm{T}}\mathbf{y}.$$

Invasion conditions for the brood masculinizer expressed by mothers are given in Figure Supplementary Figure S2C.

S6 Invasion by a maternal brood feminizer

Mothers carrying the ff genotype produce offspring who determine their own sex according to the sex allocation strategy (s_1, s_2). By contrast, mothers carrying the $F_{\mathrm{m}}f$ genotype override any sex allocation strategy by the offspring and produce only daughters. The column vector $\mathbf{x} = [x_1, \ldots, x_5]^{\mathrm{T}}$ describes the frequencies of the different types of individuals, with x_1 and x_2 describing the frequencies of ff and $F_{\mathrm{m}}f$ females breeding in environment 1 and similarly, x_3 and x_4 describing the frequencies of ff and $F_{\mathrm{m}}f$ females in environment 2. Lastly, x_5 reflects the frequencies of males that either bear genotypes ff. Note that $F_{\mathrm{m}}f$ males do not exist, as the F_{m} genotype is exclusively transmitted from mothers to daughters. The 5×5 transition matrix $\mathbf{B}$ is then given by

$$\mathbf{B} = \frac{1}{2\lambda} \times$$
$$\begin{bmatrix} pvg_{ff}(\tilde{s}_1)(1-\hat{s}_1) & \frac{1}{2}pvg_{F_{\mathrm{m}}f} & pg_{ff}(\tilde{s}_2)(1-\hat{s}_2) & \frac{1}{2}pg_{F_{\mathrm{m}}f} & p\left[vq_1 g_{ff}(\tilde{s}_1)(1-\hat{s}_1)+q_3 g_{ff}(\tilde{s}_2)(1-\hat{s}_2)+\frac{1}{2}(q_2v+q_4)g_{F_{\mathrm{m}}f}\right] \\ 0 & \frac{1}{2}pvg_{F_{\mathrm{m}}f} & 0 & \frac{1}{2}pg_{F_{\mathrm{m}}f} & \frac{1}{2}p(q_2v+q_4)g_{F_{\mathrm{m}}f} \\ (1-p)vg_{ff}(\tilde{s}_1)(1-\hat{s}_1) & \frac{1}{2}(1-p)vg_{F_{\mathrm{m}}f} & (1-p)g_{ff}(\tilde{s}_2)(1-\hat{s}_2) & \frac{1}{2}(1-p)g_{F_{\mathrm{m}}f} & (1-p)\left[vq_1 g_{ff}(\tilde{s}_1)(1-\hat{s}_1)+q_3 g_{ff}(\tilde{s}_2)(1-\hat{s}_2)+\frac{1}{2}(q_2v+q_4)g_{F_{\mathrm{m}}f}\right] \\ 0 & \frac{1}{2}(1-p)vg_{F_{\mathrm{m}}f} & 0 & \frac{1}{2}(1-p)g_{F_{\mathrm{m}}f} & \frac{1}{2}(1-p)(q_2v+q_4)g_{F_{\mathrm{m}}f} \\ g_{ff}(\tilde{s}_1)\hat{s}_1 & 0 & g_{ff}(\tilde{s}_2)\hat{s}_2 & 0 & q_1 g_{ff}(\tilde{s}_1)\hat{s}_1+q_3 g_{ff}(\tilde{s}_2)\hat{s}_2 \end{bmatrix} \tag{S22}$$

where $g_{ff}(\tilde{s}_i)$ and $g_{F_{\mathrm{m}}f}$ are the fecundities of mothers bearing the ff or $F_{\mathrm{m}}f$ genotypes. Note that $g_{ff}(\tilde{s}_i)$ is dependent on the average brood sex allocation strategy $\tilde{s}_i$, whereas $g_{F_{\mathrm{m}}f}$ is independent of any offspring sex allocation strategy, as exclusively daughters are produced:

$$g_{ff}(\tilde{s}_i) = \frac{1}{\tilde{s}_i c + 1 - \tilde{s}_i}, \quad g_{F_{\mathrm{m}}f} = 1.$$

Next, $q_k = x_k/x_5$ reflect the probabilities that a given male secures a mating with a female bearing genotype $k \in \{1,2,3,4\}$. The change in frequency $f(F_{\mathrm{m}})$ of the offspring feminizer is then given by

$$f(F_{\mathrm{m}}) = x_2 \frac{\sum_{j=1}^{5} a_{2j}x_j}{\sum_{i=1}^{5}\sum_{j=1}^{5} x_i a_{ij} x_j} + x_4 \frac{\sum_{j=1}^{5} a_{4j}x_j}{\sum_{i=1}^{5}\sum_{j=1}^{5} x_i a_{ij} x_j}. \tag{S23}$$

where the coevolving offspring sex allocation strategies follow a dynamic given by the selection differentials in eq. (S21). Consequently, invasion conditions for the brood feminizer expressed by mothers are given in Figure Supplementary Figure S2D.

S7 Errors in environmental perception

We alter the model in the main text by assuming that mothers accurately assess the state of the environment or their condition with probability $1-\varepsilon$, whereas with probability ε, mothers incorrectly perceive the opposite environment. For a mutant mother with sex-allocation strategy $(\hat{s}_i, \hat{s}_j)$, the functions f_i and m_i in eq. (A1) that give the number of her sons and daughters are now given by

$$\begin{aligned} f_i\left(\hat{s}_i, \hat{s}_j\right) =& (1-\varepsilon)\frac{1-\hat{s}_i}{\hat{s}_i c+1-\hat{s}_i}+\varepsilon\frac{1-\hat{s}_j}{\hat{s}_j c+1-\hat{s}_j}, \\ m_i\left(\hat{s}_i, \hat{s}_j\right) =& (1-\varepsilon)\frac{\hat{s}_i}{\hat{s}_i c+1-\hat{s}_i}+\varepsilon\frac{\hat{s}_j}{\hat{s}_j c+1-\hat{s}_j},. \end{aligned} \tag{S24}$$

Regarding offspring control over environmental perception, we assume that each offspring is able to perceive the particular environment individually. As a result, $1-\varepsilon$ of all offspring correctly perceive the environment, whereas ε of all offspring perceive the wrong environment, leading to the following functions f_i and m_i:

$$\begin{aligned} f_i\left(\hat{s}_i, \tilde{s}_i, \hat{s}_j, \tilde{s}_j\right) =& \frac{(1-\varepsilon)(1-\hat{s}_i)+\varepsilon\left(1-\hat{s}_j\right)}{(1-\varepsilon)\left[1-\tilde{s}_i+\tilde{s}_i c\right]+\varepsilon\left[1-\tilde{s}_j+\tilde{s}_j c\right]}, \\ m_i\left(\hat{s}_i, \tilde{s}_i, \hat{s}_j, \tilde{s}_j\right) =& \frac{(1-\varepsilon)\hat{s}_i+\varepsilon\hat{s}_j}{(1-\varepsilon)\left[1-\tilde{s}_i+\tilde{s}_i c\right]+\varepsilon\left[1-\tilde{s}_j+\tilde{s}_j c\right]}. \end{aligned}$$

In the presence of perception errors, selection differentials on s_1 and s_2 expressed in the mother are given by

$$\begin{aligned} \left.\frac{\mathrm{d}W}{\mathrm{d}\hat{s}_1}\right|_{\hat{s}_1=s_1,\hat{s}_2=s_2} =& \frac{1}{2\lambda\gamma_1^2}\left[((1-\varepsilon)y_{\mathrm{f1}}+\varepsilon y_{\mathrm{f2}})z_{\mathrm{m}}-c\left(y_{\mathrm{f2}}\varepsilon+y_{\mathrm{f1}}\left(1-\varepsilon\right)v\right)\left(z_{\mathrm{f1}}p+z_{\mathrm{f2}}\left(1-p\right)\right)\right], \\ \left.\frac{\mathrm{d}W}{\mathrm{d}\hat{s}_2}\right|_{\hat{s}_1=s_1,\hat{s}_2=s_2} =& \frac{1}{2\lambda\gamma_2^2}\left[((1-\varepsilon)y_{\mathrm{f1}}+\varepsilon y_{\mathrm{f2}})z_{\mathrm{m}}-c\left(y_{\mathrm{f2}}\varepsilon+y_{\mathrm{f1}}\left(1-\varepsilon\right)v\right)\left(z_{\mathrm{f1}}p+z_{\mathrm{f2}}\left(1-p\right)\right)\right]. \end{aligned} \tag{S25}$$

and similar selection gradients can be derived for both cases of offspring control. Results are more tedious in comparison to the error-free scenario, so we focus on a numerical analysis, corroborated with individual-based simulations (see discussion in main text and Figure S6).

S8 Supplementary Figures

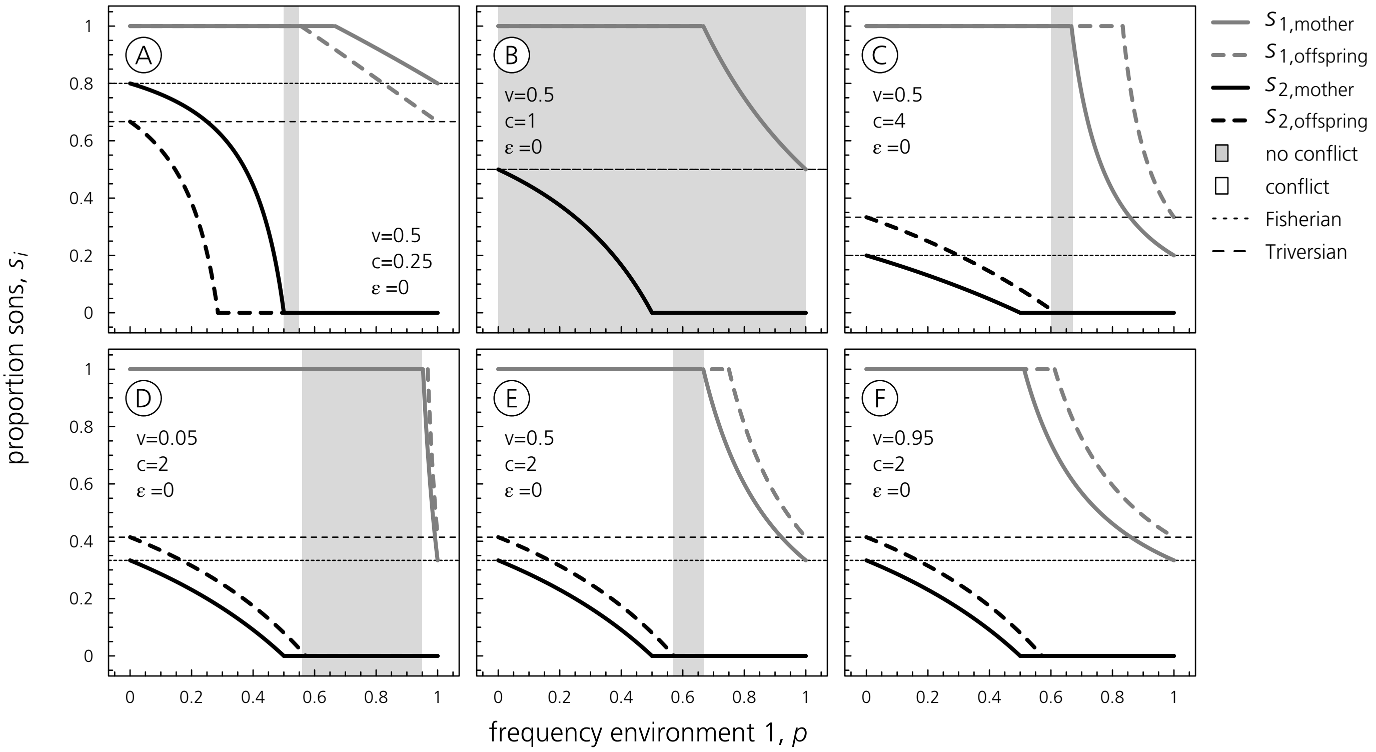

Figure S1: A comparison of maternal ($s_{1\text{m}}, s_{2\text{m}}$) (thick solid lines) and offspring ($s_{1\text{o}}, s_{2\text{o}}$) (thick dashed lines) sex-allocation strategies from Table 1 when varying survival of daughters in environment 1, v (panels A-C) and the cost of producing sons versus daughters c (panels D-F). White regions: parent-offspring conflict over sex allocation in one or both environments. Grey regions: no parent-offspring conflict over sex allocation. Thin dotted lines depict the Fisherian sex ratio $1/(1+c)$ where sex allocation is expressed in the mother and environmental variation is absent. Similarly, Triversian sex ratios $1/(1+\sqrt{c})$ are given by the thin dashed lines.

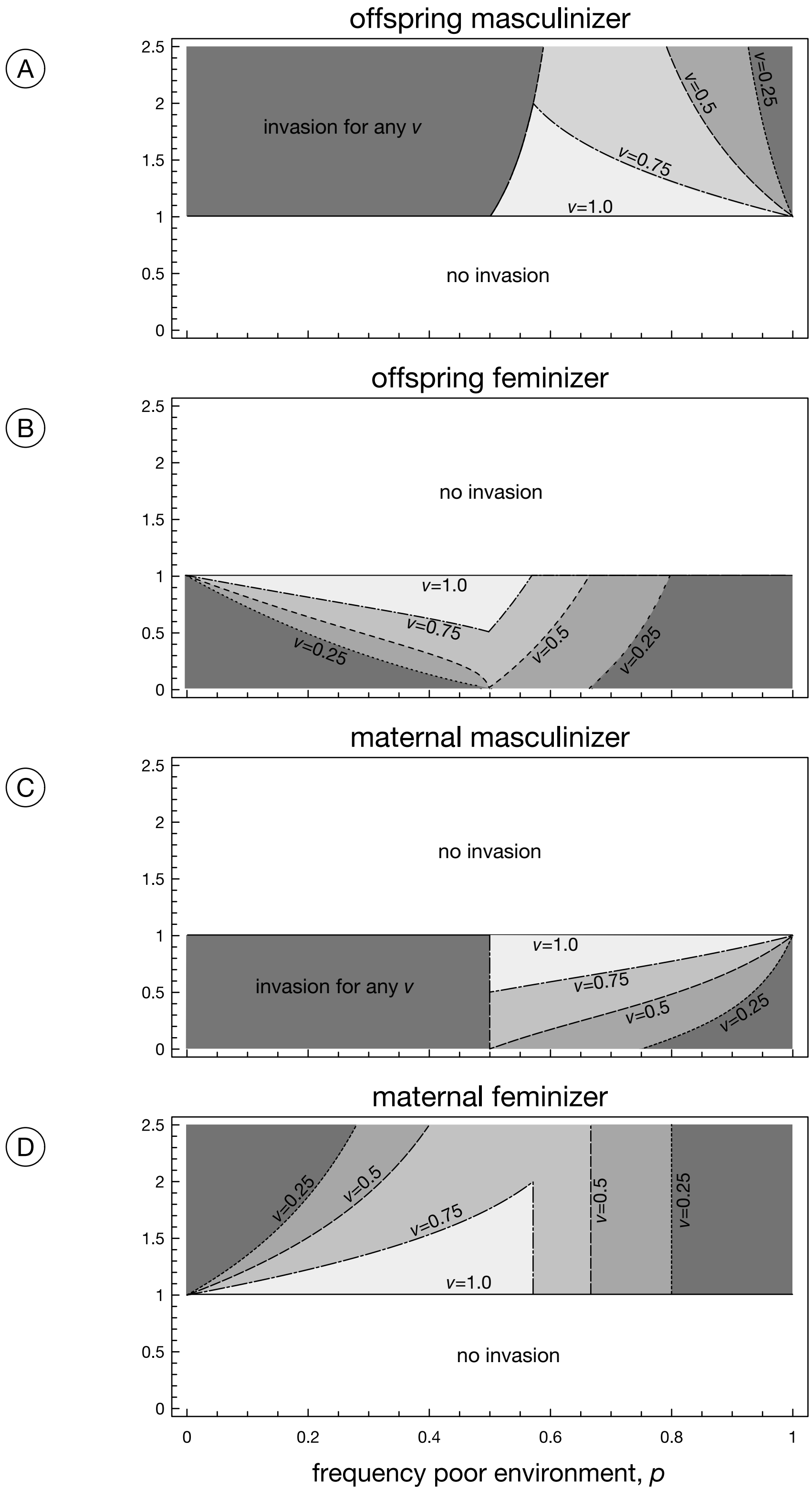

Figure S2: Summary of the analytically obtained invasion conditions for environment-independent masculinizers (panel A) and feminizers (panel B) expressed in the zygote when condition-dependent sex allocation is controlled by the mother. Similarly, panels C, D depict the invasion conditions for environment-independent masculinizers (panel C) and feminizers (panel D) expressed in the mother when condition-dependent sex allocation is controlled by the offspring. Isoclines depict the parameter space for which invasion occurs different survival probabilities v of females born in the poor environment.

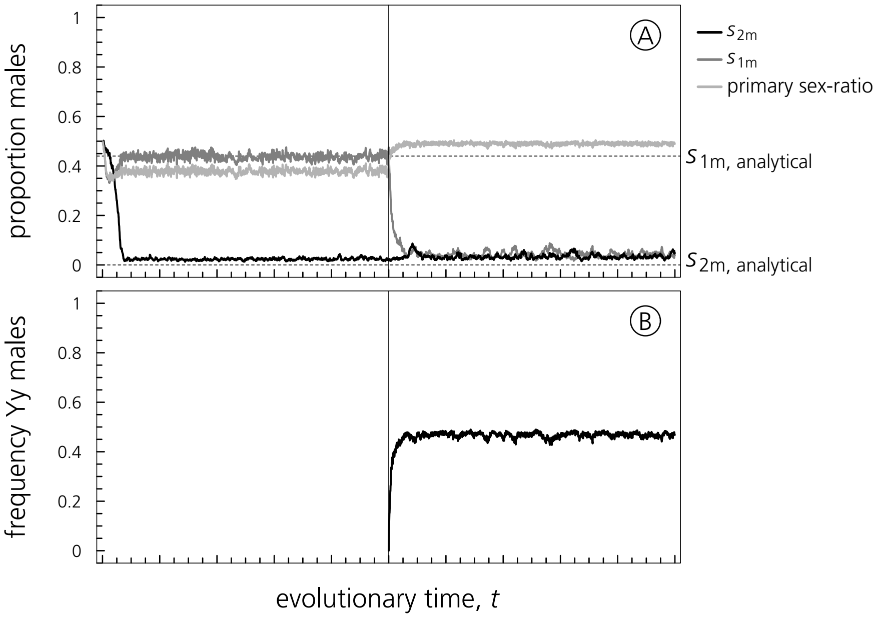

Figure S3: Example individual-based simulation showing the successful invasion by a dominant masculinizer Y expressed in the zygote. Panel A: before the invasion by Y, maternal condition-dependent sex allocation loci s_{1m} and s_{2m} achieve their analytically predicted optima (dotted lines). The subsequent invasion by Y (panel B) leads to the presence of Yy males, which selects mothers to induce those offspring who did not receive the Y chromosome to develop exclusively as daughters ($s_{1m} \to 0, s_{2m} \to 0$). As a result, sex ratios produced become independent of the environment and determined by the presence of Y only (GSD). Parameters: $c = 2,\ p = 0.9,\ v = 0.5$.

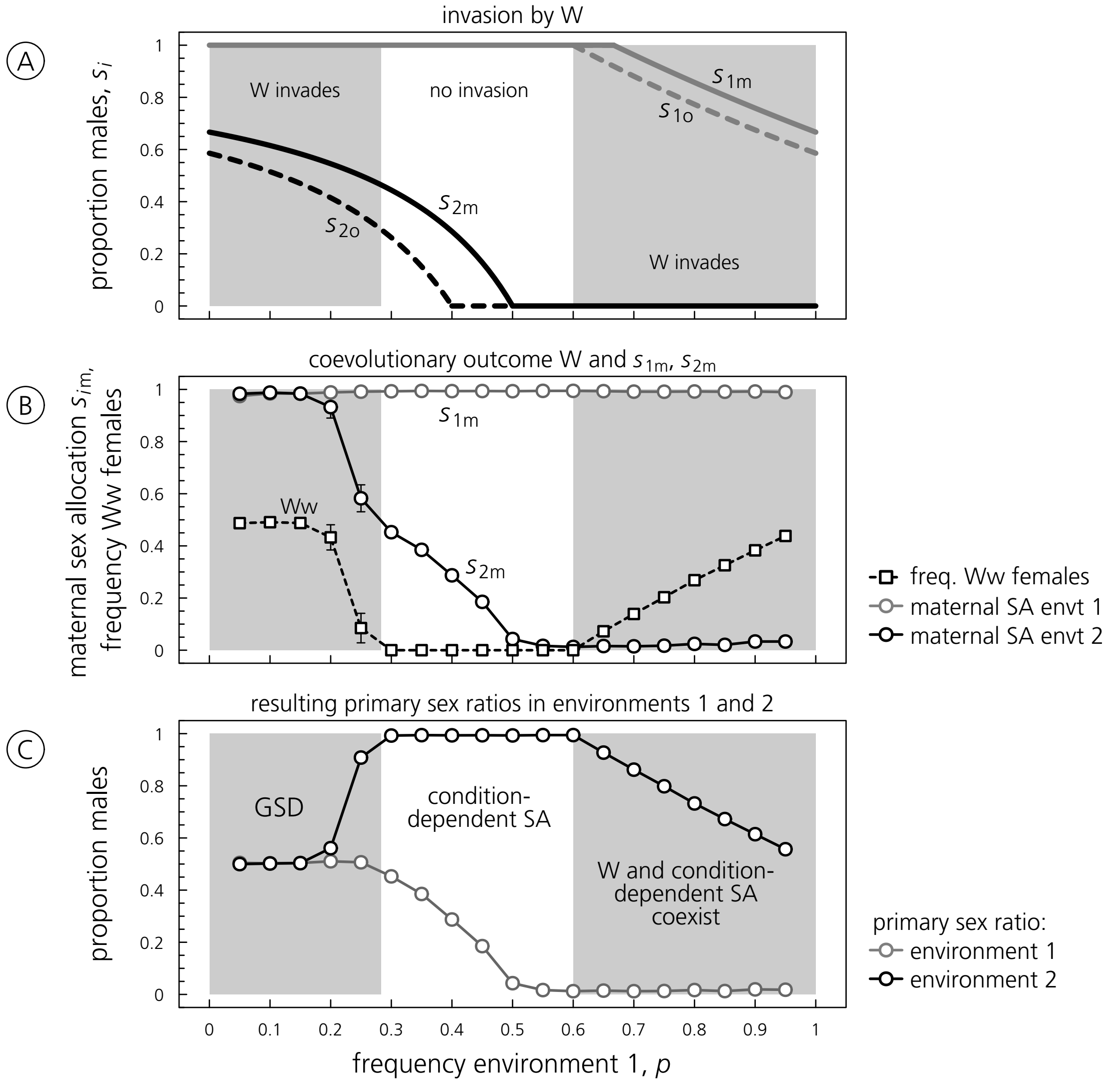

Figure S4: Invasion by a dominant feminizer (W) expressed in the zygote, when condition-dependent sex allocation is controlled by the mother (s_{1m}, s_{2m}) and when daughters are twice as costly as sons ($c = 0.5$). Panel A: grey regions depict the analytically obtained invasion conditions of W, which occurs when sex allocation optima diverge between parents and offspring. Under certain conditions, however, (left part of middle white area) sex ratio selection prevents the invasion by W despite divergent maternal and offspring sex ratio optima. Panel B: upon successful invasion, the stable frequency of W depends on the frequency p of the poor environment. As a coevolutionary response to the offspring's W, maternal sex-allocation strategies become more extreme, often leading either to $(s_{1m}, s_{2m}) \approx (1, 1)$ or $(s_{1m}, s_{2m}) \approx (1, 0)$, as shown by individual-based simulations. Panel C: primary sex ratios in both environments taken from individual-based simulations. Despite the invasion by W, the proportion of males produced still depends strongly on the environment for a substantial range of p, although resulting sex ratios are now closer to offspring than to parental optima. Only when the poor environment is relatively rare (i.e., $r < 0.28$) does the invasion by W lead to a replacement of condition-dependent sex allocation with GSD. Parameters: $v = 0.5$.

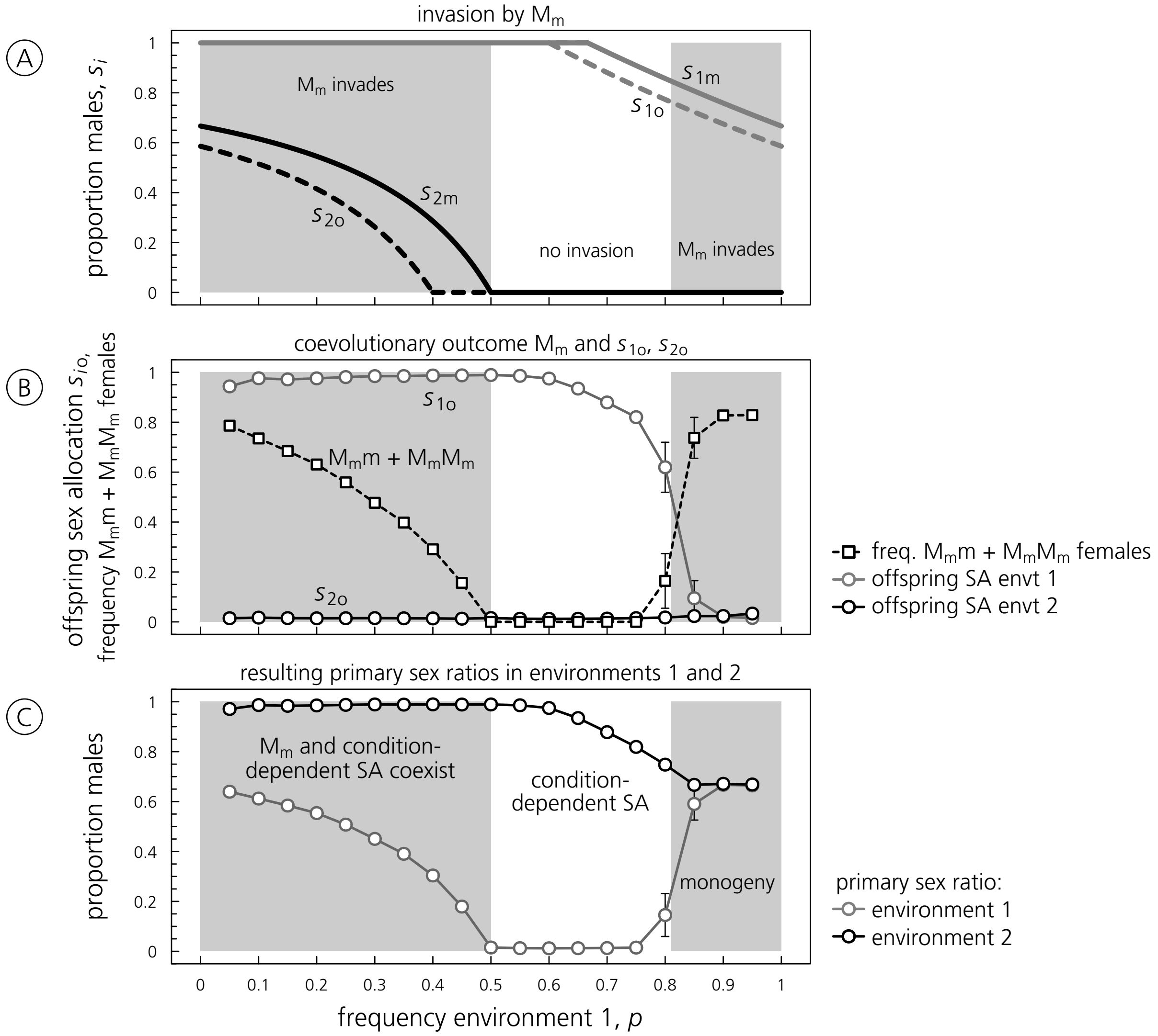

Figure S5: Invasion by a dominant masculinizer M_m expressed in the mother, when condition-dependent sex allocation is controlled by the offspring (s_{1o}, s_{2o}) and when daughters are twice as costly as sons $(c = 0.5)$. Panel A: grey areas depict the analytically obtained invasion conditions of M_m (see Figure S2C). Invasion of M_m occurs when sex allocation optima diverge between parents and offspring, but under certain conditions (right part of middle white area) sex ratio selection prevents the invasion by M_m despite divergent maternal and offspring sex ratio optima. Panel B: individual based simulations showing the coevolutionary outcome between M_m and offspring sex allocation. Upon successful invasion, the stable frequency of M_m depends on the frequency p of the poor environment. As a coevolutionary response to maternal M_m, offspring sex-allocation strategies become more extreme, often leading either to $(s_{1o}, s_{2o}) \approx (1,0)$ or $(s_{1o}, s_{2o}) \approx (0,0)$. Panel C: individual-based simulations showing the resulting primary sex ratios. Despite the invasion by M_m, condition-dependent sex allocation persists over a substantial range of p, although resulting sex ratios are now closer to parental than to offspring optima. Only when the poor environment is very common (i.e., $p > 0.8$) does the invasion by M_m lead to a replacement of condition-dependent sex allocation with monogeny, with Fisherian sex ratios $s = 1/(1 + c) = 0.67$. Parameters: $v = 0.5$.

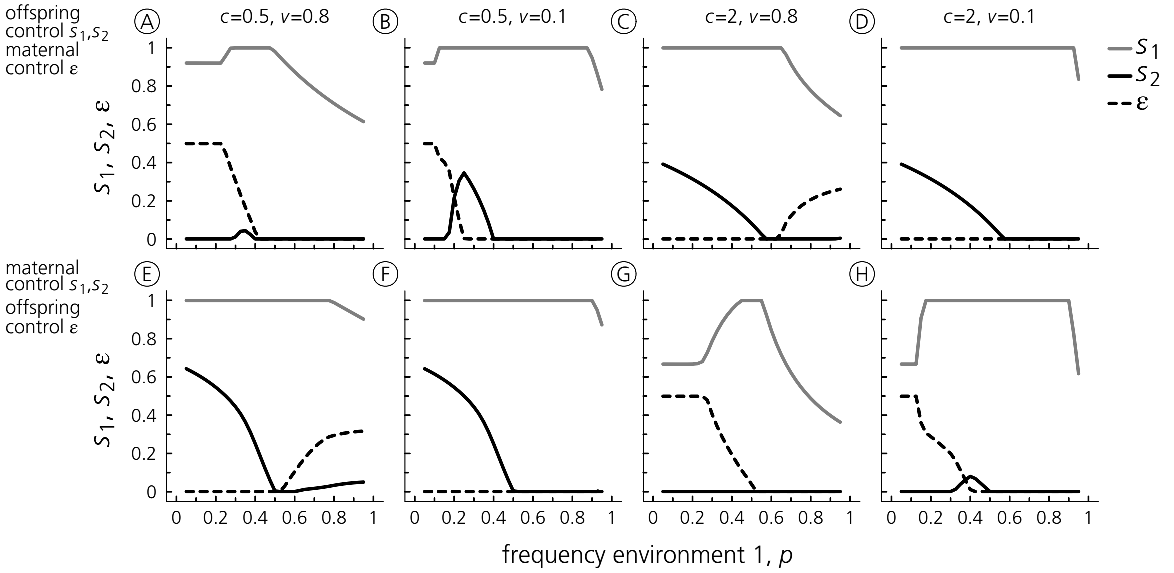

Figure S6: Coevolution between perception errors ε and condition-dependent sex allocation loci (s_1, s_2) resulting from numerical iterations of the analytical model. Panels A-C: offspring control sex allocation, whereas mothers control perception errors. Panels D-F: mothers control sex allocation, whereas offspring are in control over perception errors (e.g., by expressing hormones that may affect maternal behaviour).
